# An Amphotericin B Derivative Equally Potent to Amphotericin B and with Increased Safety

**DOI:** 10.1371/journal.pone.0162171

**Published:** 2016-09-28

**Authors:** Armando Antillón, Alexander H. de Vries, Marcel Espinosa-Caballero, José Marcos Falcón-González, David Flores Romero, Javier González–Damián, Fabiola Eloísa Jiménez-Montejo, Angel León-Buitimea, Manuel López-Ortiz, Ricardo Magaña, Siewert J. Marrink, Rosmarbel Morales-Nava, Xavier Periole, Jorge Reyes-Esparza, Josué Rodríguez Lozada, Tania Minerva Santiago-Angelino, María Cristina Vargas González, Ignacio Regla, Mauricio Carrillo-Tripp, Mario Fernández-Zertuche, Lourdes Rodríguez-Fragoso, Iván Ortega-Blake

**Affiliations:** 1 Instituto de Ciencias Físicas, Universidad Nacional Autónoma de México, Apartado Postal 48-3, 62251, Cuernavaca, Morelos, México; 2 Centro de Investigaciones Químicas, Universidad Autónoma del Estado de Morelos, Av. Universidad 1001, Col. Chamilpa Cuernavaca, Morelos, México; 3 Facultad de Farmacia, Universidad Autónoma del Estado de Morelos, Av. Universidad 1001, Col. Chamilpa Cuernavaca, Morelos, México; 4 Laboratorio Nacional de Genómica para la Biodiversidad, Centro de Investigación y de Estudios Avanzados del Instituto Politécnico Nacional, Unidad Irapuato, km 9.6 Libramiento Norte, Carretera Irapuato-León, Irapuato, Guanajuato 36821, México; 5 Departamento de Física, Centro de Investigación y de Estudios Avanzados del Instituto Politécnico Nacional, Unidad Mérida. Km 6, Carretera Antigua a Progreso, Cordemex, 97310, Mérida, Yucatán, México; 6 Groningen Biomolecular Sciences and Biotechnology Institute & Zernike Institute for Advanced Materials, University of Groningen, Nijenborgh 7, 9747 AG Groningen, The Netherlands; 7 Facultad de Estudios Superiores Zaragoza, Universidad Nacional Autónoma de México, Batalla del 5 de Mayo y Fuerte de Loreto México DF, 09230, México City, México; US Geological Survey, UNITED STATES

## Abstract

Amphotericin B is the most potent antimycotic known to date. However due to its large collateral toxicity, its use, although long standing, had been limited. Many attempts have been made to produce derivatives with reduced collateral damage. The molecular mechanism of polyene has also been closely studied for this purpose and understanding it would contribute to the development of safe derivatives. Our study examined polyene action, including chemical synthesis, electrophysiology, pharmacology, toxicology and molecular dynamics. The results were used to support a novel Amphotericin B derivative with increased selectivity: L-histidine methyl ester of Amphotericin B. We found that this derivative has the same form of action as Amphotericin B, i.e. pore formation in the cell membrane. Its reduced dimerization in solution, when compared to Amphotericin B, is at least partially responsible for its increased selectivity. Here we also present the results of preclinical tests, which show that the derivative is just as potent as Amphotericin B and has increased safety.

## Introduction

Polyene antibiotics have been used for over six decades, mainly as therapeutics for antimycotic purposes, as well as in the treatment of other ailments produced by a number of protozoa and viruses [1]. The proposed mechanism of action is the formation of pores in the cell membrane, even if other mechanisms, such as inhibition of the fungal proton ATPase, lipid peroxidation and apoptotic-like responses, have also been described [2,3]. In spite of it being a long-standing proposal, there is still ongoing controversy regarding how polyenes work to produce these pores. The transmembrane ion conductance produced is clear, as shown by electrophysiological experiments [4,5]. This is also true for other simple molecules, as natural peptides [6] that produce this effect. The standard model, proposed a long time ago [4,5], states that a barrel of polyene molecules, in particular Amphotericin B (AmB)—the most typical of these antibiotics—, forms a hydrophilic pore with the hydrophobic chain of the polyene embedded in the lipid membrane. This 'standard' model has been extended to propose that the barrel structure is stabilized by interactions between adjacent monomers, with the membrane phospholipids and sterols contained in the grooves between AmB monomers. The fact that polyenes present greater activity in cells containing ergosterol (fungi) than cholesterol (mammalian) confers them “selectivity” and enables their therapeutic use. The standard model suggests that this is due to a better interaction of ergosterol with the polyene, which leads to greater stability of the pores thus formed, according to said model. However, there is ample contradictory evidence as reviewed in Récamier *et al*. [7] and González-Damián *et al*. [8]. An alternative model, based on the presence of channels in sterol-free membranes [9–13], has been proposed [11,13]. This discussion is focused on one mechanism proposed for the action of polyenes: the formation of membrane pores. There are however other mechanisms, such as oxidative cell damage (Bratjberg et al. [14] and Sokol-Anderson [15]), destabilization of the membrane (de Kruijff and Demel [16] and Mouri et al. [17]) and the recently developed sterol extraction by clusters of the polyene: the sponge model (Palacios et al. [18)] and Gray et al. [19]).

The sponge model has also lead to the design of a novel derivative presenting greater selectivity [20]. Two recent studies, one using chemical synthesis [18] and another one using molecular dynamics [21], support this model. The first one has suggested that the mycosamine ring interacts with the sterols in a differentiated manner and thus produces selectivity. The lack of activity when mycosamine was deleted, a previously known fact [22], is taken as evidence for this. In the second study, the binding free energy between AmB and both sterols in a 1,2-dimyristoyl-*sn*-glycero-3-phosphocholine (DMPC) membrane shows a greater affinity for ergosterol over cholesterol, a requirement for the sponge model. This has been supported by a recent work using neutron reflectometry, which observes the deposition of large aggregations of AmB, but in supported bilayers and after a very large amount of AmB vs. lipid concentration [23]. Both works have contributed to the understanding of the mechanism of action, although they disregard strong evidence; e.g. in absence of sterols, the polyene channels have the same characteristics as those observed by single channel analysis in a sterol containing membrane [7,8,11,13]. Also, recent results show that the activity of Nystatine, a close analog of AmB, has a high correlation with the phase diagram of POPC/sterol mixtures [8]. Given the same amount of sterol, different activities are observed for the distinct liquid phases. Moreover, there are conditions in the phase diagram where reversed selectivity is observed, i.e., a greater activity in cholesterol over ergosterol-containing membranes. The same phenomenon was observed with AFM microscopy but only in ergosterol-containing supported lipid bilayers [24]. Several models have been recently revised by Kamiski et al. [25]. We have focused on the pore model because it allows for an explanation of the increment in selectivity attained by a new derivative.

Additional interest in understanding the mode of action of polyenes lies in their importance for therapeutic treatment. A number of commercial products have already improved on the use of AmB by reducing its collateral toxicity, such as a lipid complex like Abelcet^®^, a liposomal formulation like AmBisome^®^ and, more recently, the proposal for polymeric nanoparticles and nanosuspensions [26]. There are indeed advantages in the use of these presentations, but remnant toxicity still hinders their use, in addition to the fact that these presentations considerably increase treatment costs and could reduce the efficacy of the drug [27]. Thus more selective chemical derivatives are required, i.e., derivatives that improve the effectiveness of the molecular function, leading to more cost-effective alternatives that could in due course be considered for special delivery systems.

This is a multidisciplinary study has furthered the understanding of AmB’s modes of action. It considers other recent derivatives [21,28,29] that have shown reduced collateral toxicity towards mammalian cells, and presents a new derivative with a considerable advantage in this regard [30]. The derivative design is based on the idea that selectivity is related to membrane structure. We have therefore considered amide substitutions in the carboxylic group in order to force the sugar ring towards the membrane. The idea is that this moiety, which is essential for drug activity [23], will sense the membrane structure as well as the amides themselves. This idea could also be related to the proposed hypothesis [31] that cholesterol-containing membranes require dimerization for AmB to incorporate it. Chemical derivatives were tested on membrane patches to check their ability to produce K^+^ leakage and in microbiological studies to determine their selectivity. A novel derivative with increased safety was thus obtained. This derivative was subjected to molecular dynamics, electrophysiological, pharmacological and spectrophotometric studies in order to understand the basis of the increased selectivity. Preclinical trials were also undertaken to establish its increased safety.

## Materials

All chemicals were obtained > 95% pure from commercial suppliers and used as received unless otherwise stated. POPC dissolved in chloroform was purchased from Avanti Polar Lipids (Alabaster, AL). Powdered lecithin, cholesterol, ergosterol and AmB for pharmacological tests were purchased from Sigma-Aldrich (Toluca, Mexico), stored at -20 C (4 C for AmB) under vacuum and used without further purification. Stock chloroform solutions for every lipid were prepared once a week and stored at -20 C. AmB was purchased from Indofine Chemical Company Inc. (Hillsborough, NJ). Abelcet^®^ suspension was purchased from Armstrong Laboratories (DF Mexico). Sealed vials of ergosterol dissolved in chloroform were purchased from Supelco (Bellefonte, PA) and stored at 4 C in the dark. Dimethyl sulfoxide (DMSO, synthesis grade), potassium chloride (KCl, ACS grade) and calcium chloride (CaCl_2_, ACS grade) were purchased from Merck (Naucalpan, Mexico). Dubelcco’s PBS was purchased from Caisson Labs (North Logan, UT). All organic solvents were ACS grade and were purchased from J. T. Baker (Xalostoc, Mexico). Evaporation of the solvents was done in a rotative evaporator (B-177; Büchi Labortechnik, Flawil, Switzerland). UV spectra were obtained in a Hitachi U-5100 Spectrophotometer. Borosilicate glass capillaries were obtained from World Precision Instruments (Sarasota, FL). High-purity nitrogen gas was supplied by Praxair (Cuernavaca, Mexico). Other chemicals were purchased from Sigma-Aldrich (Toluca, Mexico). Balb-C mice (20–23 g) were purchased from Harlan México, S.A. de C.V. Blood samples were obtained from the blood bank of Instituto Nacional de Cancerología, México, to avoid the risk of transmission of infectious agents.

## Methods

### Chemical Synthesis

#### Chemistry General Procedures

**A1**-**A7** derivatives did not show an increased fungal/mammal selectivity and therefore no precise determination of purity was performed. However, since they have molecular weights above 1000 atomic units and the high-resolution mass spectroscopy measurements compare well with the calculated ones (Table 1), we were able to estimate a degree of purity of ~ 90%. All reactions using moisture and/or air-sensitive reagents were carried out in oven-dried glassware under nitrogen atmosphere; reactions involving Amphotericin B were protected from light. Thin Layer Chromatography (TLC) was run on Aldrich silica gel plates l.t. 200 μm on aluminum foil, and compounds were visualized using both/either ninhydrin solution and UV light. ^1^H NMR spectra were recorded on Inova Varian (400 and 700 MHz) instruments. Resulting data were tabulated in the following order: chemical shift (δ), multiplicity (br, broad; s, singlet; d, doublet; t, triplet; q, quartet; dd, doublet of doublets; m, multiplet), coupling constant(s) *J* (Hz), number of protons and assignation. Tetramethylsilane was used as internal reference in CDCl_3_ (δ_H_ = 0). When using DMSO-*d*_*6*_ or D_2_O, the solvent residual peak (δ_H_ = 2.50 and 4.80 ppm, respectively) was used as internal reference. ^13^C NMR spectra were recorded in the same instruments using the central signals of CDCl_3_ and DMSO-*d*_*6*_ (δ_C_ = 77.16 and 39.52 ppm, respectively) as reference signals. High and low resolution MS data were obtained on a JEOL MStation JMS-700. IR spectrum data was obtained on a Bruker Vector 22 FT-IR instrument. Melting points were determined in capillary tubes and are uncorrected. The analytical data obtained for known compounds agree with those previously reported for these compounds.

**Table 1:** Post-purification yields, representative Infrared signals and High Resolution Mass Spectrometry obtained in the synthesis of AmB amide analogues.

Analogue	Yield (%)	IR bands (cm^-1^)		M/Z	
		C = O (acid)	C = O (amide)	Calculated	Measured*
**AmB**	--	1711.0	--	923.4878	924.4930
***N*-benzylamide of AmB (A1)**	95.50	N. O.	1645.4	1012.5508	1013.6540
***N*-cyclohexylamide of AmB (A2)**	88.90	N. O.	1640.9	1004.5821	1005.6092
***N*, *N*-diisopropylamide of AmB (A3)**	93.11	N. O.	1642.8	1006.5977	1007.6423
***N*-(*S*)-*α*-phenylethylamide of AmB (A4)**	99.00	N. O.	1631.3	1026.5664	1027.5898
***N*-(*R*)-*α*-phenylethylamide of AmB (A5)**	98.45	N. O.	1630.1	1026.5664	1027.5785
***N*-(*L*)-tryptophanamide of AmB (A6)**	65.13	N. O.	1635.4	1123.5828	1124.5276
***N*-(*D*)-tryptophanamide of AmB (A7)**	93.85	N. O.	1638.2	1123.5828	1124.6073
***L*-histidine methyl ester of AmB (A21)**	84.90	N. O.	1652.38	1075.5702	1075.5719

* Measured values are presented as [M + H]^+^

#### Synthesis of Amphotericin B amide analogues (A1–A7)

**Fig 1:**
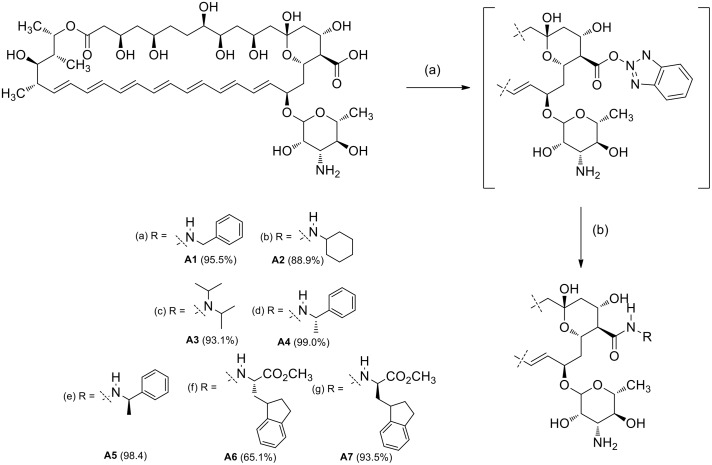
Synthesis of Amphotericin B amide analogues A1–A7. Reagents and conditions: (a) Selected amine, DPPA, Et_3_N, DMAC, rt.

Et_3_N (5.0 mMol), DPPA (5.0 mMol) and the selected amine (5.0 mMol) were added to a stirred solution of Amphotericin B (0.5 mMol) in 10 ml of DMAC [32] under nitrogen atmosphere (Fig 1). The reaction mixture was stirred at room temperature (rt) until complete consumption of the starting materials (TLC system: methanol–chloroform–water 20:10:1 v/v). The product was precipitated with anhydrous diethyl ether, dissolved in *n*-BuOH and washed with water (2 x 50 ml). The solvent was evaporated at reduced pressure. The product was precipitated and subsequently washed with anhydrous diethyl ether (3 x 50 ml) and hexanes (1 x 50 ml).

**Analogue A1, *N*-benzylamide of AmB:** This analogue was obtained in yield 34% and isolated as a yellow solid with mp 187 C (dec). ^1^H (700 MHz, DMSO-_d-6_): δ 7.29–7.21 (m, 5 H), 6.45–5.94 (m, 12 H), 5.83 (s, 1 H), 5.46–5.39 (m, 2 H), 5.20 (br, 1 H), 4.77–4.72 (m, 2 H), 4.45–4.22 (m, 6 H), 4.04–3.98 (m, 3 H), 3.76–3.45 (m, 6 H), 3.22 (s, 1 H), 3.15–3.07 (m, 2 H), 2.94–2.92 (m, 1 H), 2.28 (m, 2 H), 2.16 (m, 1 H), 2.03–1.97 (m, 2 H), 1.89–1.21 (m, 16 H, CH_2_, CH), 1.12–1.09 (m, 6 H, CH_3_), 1.02 (d, *J* = 5.8 Hz, 3 H, CH_3_), 0.90 (d, *J* = 6.6 Hz, 3 H). Due the low solubility of **A1** wasn’t possible to obtain ^13^C spectra. HRMS (FAB+): [M + H]^+^ calcd for C_54_H_80_N_2_O_16_, 1013.5508; found, 1013.6540.

**Analogue A2, *N*-cyclohexylamide of AmB:** This compound was obtained in yield 54% and isolated as a yellow solid with mp 143 C (dec). ^1^H (700 MHz, DMSO-_d-6_): δ 6.45–5.92(m, 12 H), 5.81–5.76 (br, 1 H), 5.43–5.40 (m, 1 H), 5.20 (br, 1 H), 4.77–4.64 (m, 2 H), 4.45–4.36 (m, 2 H), 4.30 (s, 1 H), 4.22–4.18 (m, 2 H), 4.04–4.02 (m, 2 H), 3.62 (s, 1 H), 3.54–3.50 (m, 2 H), 3.19–3.15 (m, 1 H), 3.09–3.06 (m, 2 H), 3.00–2.95 (m, 1 H), 2.39 (d, *J* = 8 Hz, 1 H), 2.28–2.26 (m, 1 H), 2.15 (d, *J* = 4.7 Hz, 2 H), 1.97–1.95 (m, 1 H), 1.89–1.22 (m, 22 H), 1.17–1.05 (m, 9 H), 1.02 (d, *J* = 6.1 Hz, 3 H), 0.89 (d, *J* = 6.9 Hz, 3 H). ^13^C (175 MHz, DMSO-_d-6_): δ 171.30, 171.01, 137.20, 134.33, 134.13, 133.91, 133.63, 132.92, 132.87, 132.63, 132.31, 132.28, 131.65, 129.02, 97.51, 97.08, 77.60, 74.76, 74.25, 74.01, 73.65, 69.77, 69.56, 69.30, 68.20, 66.63, 65.75, 64.91, 57.16, 57.01, 47.82, 33.31, 32.82, 29.46, 25.66, 25.03, 24.91, 18.93, 18.33, 17.41, 12.52. HRMS (FAB+): m/z [M + H]^+^ for C_53_H_84_N_2_O_16_ calcd: 1005.5821, found: 1005.6092.

**Analogue A3, *N*, *N*-diisopropylamide of AmB:** This compound was obtained in yield 36% and isolated as a yellow solid with mp 140 C (dec). ^1^H (700 MHz, DMSO-_d-6_): δ 6.45–5.92 (m, 12 H), 5.44–5.40 (m, 1 H), 5.19 (br, 1 H), 4.36–4.34 (m, 1 H), 4.31 (s, 1 H), 4.21 (m, 1 H), 4.04–4.03 (m, 2 H), 3.88–3.82 (m, 1 H), 3.50 (m, 1 H), 3.10–3.06 (m, 2 H), 3.02–3.00 (m, 1 H), 2.28–2.26 (m, 1 H), 2.15 (d, *J* = 4.7 Hz, 1 H), 1.88–1.21 (m, 20 H), 1.17–1.01 (m, 15 H), 0.89 (d, *J* = 6.9 Hz, 3 H). ^13^C (175 MHz, DMSO-_d-6_): δ 171.35, 171.01, 137.23, 134.31, 134.15, 133.88, 133.63, 132.93, 132.65, 132.31, 131.65, 129.07, 97.51, 96.76, 77.55, 74.27, 74.01, 73.54, 73.49, 69.66, 69.57, 69.32, 68.22, 66.77, 66.63, 65.63, 65.00, 56.68, 30.04, 29.46, 23.09, 22.89, 18.93, 18.26, 17.42, 12.52.; HRMS (FAB+): m/z [M + H]^+^ for C_53_H_86_N_2_O_16_ calcd: 1007.5977, found: 1007.6423.

**Analogue A4, *N-(S)-α*-phenylethylamide of AmB:** This compound was obtained in yield 37% and isolated as a yellow solid with mp 161 C (dec). ^1^H (700 MHz, DMSO-_d-6_): δ 7.38–7.21 (m, 5 H), 6.47–5.96 (m, 12 H), 5.46–5.42 (m, 1 H), 5.22 (br, 1 H), 5.00 (m, 1 H), 4.43–4.38 (m, 2 H), 4.26 (m, 2 H), 4.06 (m, 2 H), 3.74 (s, 1 H), 3.52–3.47 (m, 2 H), 3.11 (m, 2 H), 2.285 (m, 1 H), 2.16 (m, 1 H), 2.04 (m, 1 H), 1.895 (m, 1 H), 1.72–1.23 (m, 15 H), 1.19–1.08 (m, 6 H), 1.04 (d, *J* = 6.1 Hz, 3 H) 0.91 (d, *J* = 6.9, Hz, 3 H). ^13^C (175 MHz, DMSO-_d-6_): δ 172.38, 171.01, 144.52, 137.24, 134.31, 134.15, 133.85, 133.62, 132.96, 132.66, 132.31, 131.61, 129.22, 128.49, 126.91, 126.65, 97.55, 96.70, 69.67, 69.56, 69.52, 69.32, 68.23, 66.63, 66.59, 65.67, 65.12, 47.76, 29.46, 22.87, 18.98, 17.43, 12.52. HRMS (FAB+): m/z [M + H]^+^ for C_55_H_82_N_2_O_16_ calcd: 1027.5664, found: 1027.5898.

**Analogue A5, *N-(R)-α*-phenylethylamide of AmB:** This compound was obtained in yield 33% and isolated as a yellow solid with mp 166 C (dec). ^1^H (700 MHz, DMSO-_d-6_): δ 7.35–7.12 (m, 5 H), 6.48–5.95 (m, 12 H), 5.49–5.40 (m, 1 H), 5.23 (br, 1 H), 5.00–4.94 (m, 1 H), 4.78–4.73 (m, 1 H), 4.36 (s, 1 H), 4.24 (m, 2 H), 4.11–3.99 (m, 2 H), 3.55–3.48 (m, 2 H), 3.24 (br, 1 H), 3.13–3.09 (m, 2 H), 2.28 (m, 1 H), 2.16 (m, 1 H), 1.89 (m, 1 H), 1.72–1.23 (m, 15), 1.13–1.10 (m, 6 H), 1.04 (d, *J* = 5.5 Hz, 3 H), 0.91, (d, *J* = 6.3 Hz, 3 H). ^13^C (175 MHz, DMSO-_d-6_): δ 172.39, 171.02, 145.13, 137.19, 134.30, 134.14, 133.73, 133.62, 132.97, 132.72, 132.66, 132.33, 131.74, 128.89, 128.85, 128.62, 128.34, 126.40, 99.12, 97.64, 74.40, 73.92, 69.66, 69.51, 69.16, 68.34, 66.59, 65.89, 64.77, 47.61, 29.49, 18.98, 18.63, 17.35, 12.56. HRMS (FAB+): m/z [M + H]^+^ for: C_55_H_82_N_2_O_16_ calcd: 1027.5664, found: 1027.5785.

**Analogue A6, *N-(L)*-tryptophanamide of AmB:** This compound was obtained in yield 60% and isolated as a yellow solid with mp 142 C (dec). ^1^H (700 MHz, DMSO-_d-6_): δ 7.98 (br, 1 H), 7.44 (d, *J* = 7.7 Hz, 1 H), 7.31 (d, *J* = 7.9 Hz, 1 H), 7.25 (s, 1 H), 7.21 (br, 1 H), 7.05 (dd, *J* = 7.9, 7 Hz, 1 H), 6.97 (dd, *J* = 7.7, 7 Hz, 1 H), 6.46–5.97 (m, 12 H), 5.80 (s, 1 H), 5.67 (d, *J* = 5.4 Hz, 1 H), 5.44–5.40 (m, 1 H), 5.34 (d, *J* = 5.2 Hz, 1 H), 5.20 (m, 1 H), 4.78–4.72 (m, 1 H), 4.69 (dd, *J* = 7.0, 7.0 Hz, 1 H) 4.52–4.47 (m, 1 H), 4.40 (br, 1 H), 4.23 (m, 1 H), 4.05–4.00 (m, 3 H), 3.81 (br, 1 H), 3.57 (s, 3 H), 3.52 (m, 1 H), 3.26–3.19 (m, 1 H), 3.14–3.03 (m, 1 H), 2.29–2.25 (m, 1 H), 2.16 (d, *J* = 5.1 Hz, 1 H), 1.89–1.87 (m, 1 H), 1.71–1.21 (m, 14 H), 1.165 (d, *J* = 6.0 Hz, 3 H), 1.13 (d, *J* = 5.6 Hz, 3 H), 1.09 (d, *J* = 6.3 Hz, 3 H). ^13^C (175 MHz, DMSO-_d-6_): δ 173.10, 172.83, 171.01, 136.46, 136.41, 134.33, 134.11, 133.87, 133.62, 132.94, 132.65, 132.30, 131.67, 127.51, 124.2, 121.36, 118.81, 118.3, 111.83, 109.78, 97.57, 95.51, 77.58, 73.07, 69.53, 69.31, 69.06, 69.02, 68.31, 67.50, 66.78, 66.62, 66.46, 65.50, 65.38, 55.95, 53.36, 52.34, 29.48, 27.21, 18.92, 18.11, 17.40, 12.52. HRMS (FAB+): m/z [M + H]^+^ calcd for: C_59_H_85_N_3_O_18_ 1124.5828, found: 1124.5276.

**Analogue A7, *N-(D)*-tryptophanamide of AmB:** This compound was obtained in yield 68% and isolated as a yellow solid with mp 146 C (dec). ^1^H (700 MHz, DMSO-_d-6_): δ 7.98 (br, 1 H), 7.44 (d, *J* = 7.7 Hz, 1 H), 7.31 (d, *J* = 7.9 Hz, 1 H), 7.25 (s, 1 H), 7.21 (br, 1 H), 7.05 (dd, *J* = 7.9, 7 Hz, 1 H), 6.97 (dd, *J* = 7.7, 7 Hz, 1 H), 6.46–5.97 (m, 12 H), 5.80 (s, 1 H), 5.67 (d, *J* = 5.4 Hz, 1 H), 5.44–5.40 (m, 1 H), 5.34 (d, *J* = 5.2 Hz, 1 H), 5.20 (m, 1 H), 4.78–4.72 (m, 1 H), 4.69 (dd, *J* = 7.0, 7.0 Hz, 1 H) 4.52–4.47 (m, 1 H), 4.40 (br, 1 H), 4.23 (m, 1 H), 4.05–4.00 (m, 3 H), 3.81 (br, 1 H), 3.57 (s, 3 H), 3.52 (m, 1 H), 3.26–3.19 (m, 1 H), 3.14–3.03 (m, 1 H), 2.29–2.25 (m, 1 H), 2.16 (d, *J* = 5.1 Hz, 1 H), 1.89–1.87 (m, 1 H), 1.71–1.21 (m, 14 H), 1.16 (d, *J* = 6.0 Hz, 3 H), 1.13 (d, *J* = 5.6 Hz, 3 H), 1.09 (d, *J* = 6.3 Hz, 3 H). ^13^C (175 MHz, DMSO-_d-6_): δ 173.10, 172.83, 171.01, 136.46, 136.41, 134.33, 134.11, 133.87, 133.62, 132.94, 132.65, 132.30, 131.67, 127.51, 124.2, 121.36, 118.81, 118.3, 111.83, 109.78, 97.57, 95.51, 77.58, 73.07, 69.53, 69.31, 69.06, 69.02, 68.31, 67.50, 66.78, 66.62, 66.46, 65.50, 65.38, 55.95, 53.36, 52.34, 29.48, 27.21, 18.92, 18.11, 17.40, 12.52. HRMS (FAB+): m/z [M + H]^+^ for C_59_H_85_N_3_O_18_ calcd: 1124.5828, found: 1124.6073.

#### Synthesis of Amphotericin B amide analogue A21

**Fig 2:**
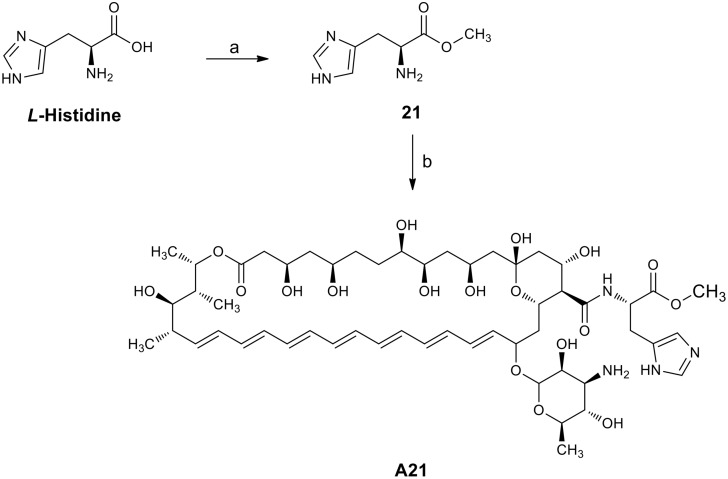
The synthesis of the A21 analogue produced from Amphotericin B. Reagents and conditions: (a) SOCl_2_, MeOH, 60°C; (b) AmB, PyBOP, Et_3_N, DMSO, rt.

**a) *L*-histidine methyl ester dihydrochloride.** SOCl_2_ (10 ml, 137 mMol) was slowly added to a stirred solution of *L*-histidine (5 g, 32.22 mMol) in 30 ml of methanol at 0 C. The reaction mixture was then heated to 60 C for 6 h (TLC system: methanol) (Fig 2). The solvent was evaporated, and the crude product recrystallized from methanol/ether [33] to obtain the *L*-histidine methyl ester dihydrochloride **21** which was used without further purification (yield 99.00%): mp 195 C; ^1^H NMR (200 MHz D_2_O) *δ* 8.72 (s, 1H, Im-*2-H*), 7.50 (s, 1H, Im-*5-H*), 4.55 (t, J = 7 Hz, 1H, NH_2_C*H*), 3.86 (s, 3H, C*H*_*3*_), 3.51 (m, 2H, C*H*_*2*_).

**Fig 3:**
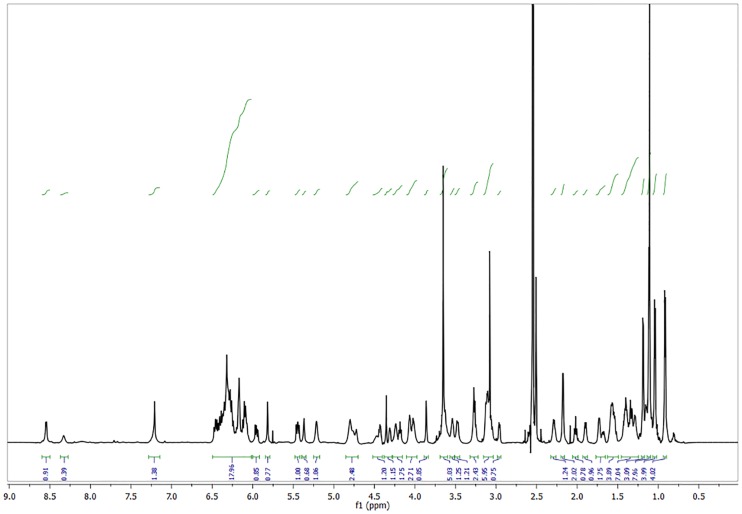
^1^H NMR spectrum of A21 analogue.

**Fig 4:**
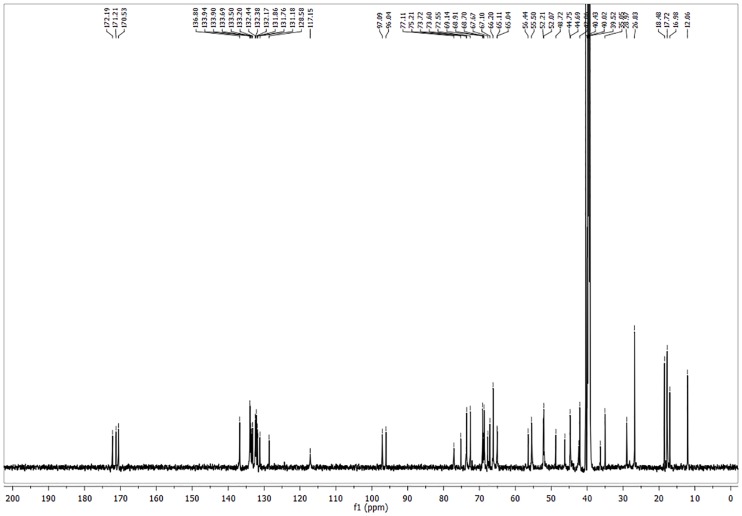
^13^C NMR spectrum of A21 analogue.

**b) Preparation of analogue A21 up to 100 mg.** Et_3_N was added drop wise to a solution of Amphotericin B (0.195 mMol) and *L*-Histidine methyl ester dihydrochloride **21** (0.409 mMol, 2.1 eq.) in DMSO until pH = 8. The resulting mixture was stirred for 15 minutes. After this, PyBOP (0.292 mMol, 1.5 eq.) was added under nitrogen atmosphere, the flask was sealed and stirred for 72 h at rt. (TLC system: methanol–chloroform–water 20:10:1 v/v). The product was precipitated and washed with anhydrous diethyl ether (5 x 5 ml) and anhydrous acetone (5 x 30 ml). The suspension obtained was centrifuged at 3500 rpm for 10 minutes. The solvent was decanted and the product dried at reduced pressure to obtain a yellowish powder corresponding to the AmB analogue **A21**. This compound was obtained in yield 84.9% and isolated as a yellow solid with mp 140–145 C (dec); ^1^H NMR (Fig 3) (700 MHz, pyridine) *δ* 8.16 (d, *J* = 12.1 Hz, 1 H, Im-*2-H*), 7.09 (s, 1 H, Im-*5-H*), 6.96–6.26 (m, 14 H, olefinic *H*), 5.89 (d, *J* = 6.8 Hz, 3 H), 5.54 (dd, *J* = 24.2, 13.8 Hz, 3 H), 5.39–5.20 (m, 4 H), 5.20–4.65 (m, 9 H), 4.49 (s, 1 H), 4.40–4.09 (m, 4H), 4.03–3.96 (m, 1H), 3.81–3.36 (m, 6H, methyl ester of L-His *H* included), 3.11–2.60 (m, 6H), 2.59–2.39 (m, 3H), 2.30–1.18 (m, 26H, aliphatic *H*), 1.17–0.95 (m, 2H), 0.93–0.77 (m, 1H). ^13^C NMR (Fig 4) (176 MHz, pyridine) δ 174.21, 173.85, 173.02, 172.21, 150.81, 150.57, 149.91, 137.66, 136.30, 136.20, 136.16, 135.60, 134.98, 134.26, 133.54, 133.26, 133.00, 124.18, 124.13, 123.57, 101.69, 98.57, 98.44, 78.84, 76.95, 75.41, 75.33, 74.98, 72.32, 70.40, 70.16, 68.68, 67.06, 66.53, 58.32, 54.56, 52.55, 47.74, 46.26, 45.62, 44.08, 43.33, 43.24, 41.42, 36.88, 32.28, 30.54, 19.38, 18.95, 18.84, 17.67, 17.64, 13.12, 9.28; HRMS (FAB^+^): m/z [M + H]^+^ for C_54_H_82_N_4_O_18_ calcd: 1075.5702, found: 1075.5719; IR vmax 3274.23

**Fig 5:**
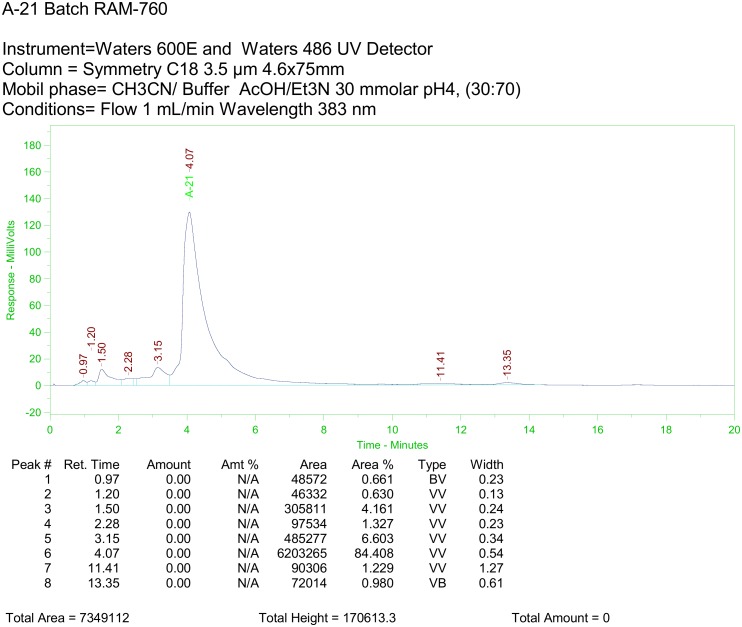
HPLC Chromatogram of A21 analogue.

**c) Preparation of analogue A21 up to 10 g**. In a 3 neck 1 L flask provided with mechanical stirrer, thermometer, input and output of nitrogen, 10 g (10.82 mMol) of AmB and 5.24 g (21.64 mMol), of *L*-Histidine methyl ester dihydrochloride in 100 ml of DMSO were loaded in the absence of light. 6.78 ml of Et_3_N (48.7 mMol) was added dropwise to the resulting slurry, which was then stirred for 15 minutes. PyBOP (11.26 g; 21.65 mMol) was added at once under nitrogen atmosphere. The flask was sealed and stirred for 12–24 h at rt., verifying the total consumption of AmB by HPLC analysis. Reaction monitoring was performed on a Waters 600 HPLC System equipped with UV 486 detector under the following parameters: Column: Symmetry C18 4.6 x 75 mm, 3.5 μm, Mobile phase: CH_3_CN/ AcOH/Et_3_N 30 mMol pH = 4 Buffer, (30:70), Injection volume: 5 μL/0.125 mg/ml, Flow: 1 ml/min and λ = 383 nm. The DMSO solution was washed with anhydrous MTBE (6 x 100 ml) and precipitated by the addition of anhydrous acetone (1 l). The suspension obtained was centrifuged at 3500 rpm for 10 minutes at 4 C. The solvent was decanted, the product washed again with anhydrous acetone (2 X 500 ml) and dried at 0.05 Torr to obtain 10.78 g (92.70%), melting point 140–145 C (dec) of a yellowish powder corresponding to the AmB analogue **A21**. m/z [M + H]^+^ for C_54_H_82_N_4_O_18_ calcd: 1075.5702, found: 1075.5719; **A21** analogue HPLC spectrum is shown in Fig 5. It was obtained with a model 600E Waters chromatograph coupled to a Waters 486 UV-Vis detector.

### Electrophysiology

Single channel studies of the membrane pore of AmB and several of its derivatives were performed using the tip-dip technique.

#### Preparation of small unilamellar vesicle

Cholesterol or ergosterol solutions were mixed with POPC to obtain the desired mol fraction of sterol (30%). The solvent was evaporated and the suspension was prepared by adding the working solution (2 M KCl, 1 mMol CaCl_2_, 10 mMol HEPES [pH 8.0]) to the film deposited in the flask and then treated in an ultrasonic bath to produce dispersion and obtain unilamellar vesicles [34]. The suspension was stored for 2 h under refrigeration (4–6 C) prior to polyene incorporation.

#### Polyene Incorporation

Powdered polyenes were stored at -20 C and used without further purification. Stock solution (5 mMol) was prepared in DMSO and used the same day. In order to homogenize, the sample in the solvent was subjected to ultrasonic dispersion. The proper amount of the stock solution of the polyene was added to the small unilamellar vesicle (SUV) preparation in order to obtain the desired concentration. Homogenized suspension was obtained via ultrasonic dispersion in an N_2_ -enriched atmosphere after adding the polyene. The antibiotic-liposome micro emulsion was then immersed in an ultrasonic bath for 15 min and stored at 4 C for 24 h before use in an N_2_ enriched atmosphere.

#### Micropipette Fabrication

Glass capillaries with filament were pulled using the P2000 instrument from Sutter Instruments (Novato, CA). Micropipettes were then filled with the same working solution used for the preparation of SUV’s and used within the following 20 min. The average resistance of micropipettes in the working solution was 100 ± 25 MΩ.

#### Solvent-Free Tip-Dip Lipid Bilayer Formation and Electrical Measurements

A sample of the SUV was put in a controlled-temperature chamber, where the oxygen excess was removed by a continuous nitrogen flux. The sample was settled for 10 min at the desired temperature, and then a bilayer was formed at the tip of the micropipette by consecutive immersion in SUV suspension until a capacitive response to the square potential applied was observed [35,36]. In all cases, the lipid concentration was 4 mg/ml. The standard seals obtained in this manner were of ~100 GΩ with a 2 kHz low pass filter and a current root mean-square I_rms_ ~ 0.25 pA. The chamber was electrically insulated with a Faraday cage and suspended in elastic bands to reduce the mechanical vibration. The current signal was amplified with an Axopatch 200B and digitally converted with a Digidata 1320, both from MDS Analytical Technologies (Toronto, Canada), and stored in a personal computer. The signal was acquired with the aid of Clampex 8.2 software from MDS Analytical Technologies at a frequency of 10 kHz. The potential applied in all experiments was 100 mV. Care had to be taken to ensure that experiments were being done in conditions of equilibrium. We determined the time average conductance as a function of time and noticed that this property needs 15 min to attain equilibrium. All records were baseline-corrected *a posteriori* using an in-house computer program. The currents here presented correspond to the average currents occurring in a 5 min experiment obtained from an all points histogram. All graphics were done with XmGrace free software.

### UV Spectrophotometry

Dimerization of the *L*-Histidine derivative and AmB was determined using the absorption spectra. UV Absorption Spectra for AmB and compound **A21** were obtained in the following manner. Aliquots of the polyene (stock solution in DMSO for AmB and in PBS for **A21**, both in N_2_ atmosphere were kept in closed vessels) were added to Dubelcco’s PBS solution at pH = 7.46 at different concentrations and used to obtain the absorption spectra in a Hitachi U-5100 spectrophotometer at rt under N_2_ atmosphere.

### Toxicological Activity

Toxicological activity of the different compounds was determined via in vitro antifungal assays, hemolysis tests, cell cultures and viability, as well as preclinical tests in mice and histopathological analysis.

#### Statistical methods

The data were represented as the mean ± SD. The data were statistically analyzed using the SPSS 10.0 software (SPSS Inc., Chicago, Ill., USA), the t-test, and ANOVA. Differences were considered significant if the p-value was less than 0.05. For disseminated candidiasis, we analyzed the results by a two-tailed Fisher’s exact test.

#### In vitro antifungal assays

The antifungal activity of the AmB and its analogs was determined by a flow cytometry method as previously described in reference [37]. For testing, we used two strains of *Candida albicans* (ATCC 10231 and 752) and *Candida krusei* (ATCC 6258). 1 x 10^6^ CFU/ml were seeded in plates of 96 wells. The cells were treated with AmB and its analog **A21** at concentrations of 0.01, 0.1, 1, 10, 100 and 1000 μM; furthermore in a zoom-in experiment we also performed a second set at concentrations of 0.1, 0.2, 0.4, 0.6, 0.8 1.0 and 10 μM; AmB was dissolved in 1% v/v DMSO and **A21** in a PBS solution with pH 7.4. We did control experiments with the DMSO at 1% v/v solution and found the following toxicities: 0.5% for *Candida albicans* (ATCC 10231), 0.6% for *Candida albicans* (ATCC 752) and 0.2% for *Candida krusei* (ATCC 6258). These values were subtracted from the respective results. Cells were incubated for 24 h at 37 C under aerobic conditions; and collected by centrifugation at 10,000 × g for 10 min. Cells were washed once in phosphate buffer solution stained with 0.1mg/ml propidium iodide (PI), and incubated for 30 minutes at rt and protected from light. Finally the samples were analyzed by flow cytometry (Becton-Dickinson Calibur Facsc, 480 nm argon laser CA, USA). The parameters and intrinsic fluorescence in the FL2 channel (fluorescent yellow/orange) for FUN and channel FL3 (red fluorescence filter, 630 nm) for PI were purchased and registered on a logarithmic scale for a minimum of 7500 events. The quadrants were defined using the fluorescence of control samples, so they include up of 5% of cells in the upper right quadrant, and then used to analyze the remaining samples to quantify the percentage of cells showing altered fluorescence compared to drug-free controls.

#### Hemolysis tests

The blood was drawn from humans into an evacuated siliconized glass tube and stirred to remove fibrinogen in the Blood Bank of Instituto Nacional de Cancerología, México. The blood was diluted with an isotonic phosphate buffer (PBS) solution with pH 7.4 and centrifuged at 2500 rpm for 15 min and the supernatant was discarded. The PBS consisted of Na_2_HPO_4_ (7.95 g), KH_2_PO_4_ (0.76 g), NaCl (7.20 g), and distilled water (1000 ml). The erythrocytes were washed until supernatant was clear, and the packed cells were resuspended in PBS buffer solution (pH 7.4) to form 2% red blood cells. The stock dispersion was stored in a refrigerator for a maximum of 48 h checking for stability by photometric monitoring. The hemolytic activities of AmB and its analogs were investigated as described by Jung *et al*., [38]. Briefly, 1 x 10^7^ cells /ml were considered. Erythrocytes were resuspended with 450 μl of a solution of 150 mM KCl + 3 mM Tris (pH 7.4). Erythrocytes were treated with AmB and its analogs and were incubated at 37 C for 1 h. Cells were treated with AmB and it analog **A21** at concentrations of 0.01, 0.1, 1, 10, 100 and 1000 μM; (AmB was dissolved in 1% v/v DMSO and **A21** in a PBS solution with pH 7.4). We did control experiments with a 1% v/v DMSO solution and found a toxicity of 28.13% for erythrocytes. This value was subtracted from all the results. Furthermore, in a zoom-in experiment, we performed a second set at concentrations of 0.1, 0.2, 0.4, 0.6, 0.8 1.0 and 10 μM (AmB and **A21** were prepared as mentioned above). After 24 h the solution was centrifuged (Beckman Instruments Inc., USA) at 3000 rpm and the supernatants were taken out. 100 μl of the supernatant was dissolved in 2 ml of an ethanol/HCl mixture to dissolve all components and avoid the precipitation of hemoglobin. The supernatant was analyzed at 398 nm by UV spectrometer (UV-1601, Shimadzu, Japan). Normal saline concentration was used as negative control (0% lysis) and distilled water as positive control (100% lysis). The hemolysis rate (HR) was calculated as follows:

$$HR\left(\%\right)=\left(\left(D_{s}-D_{nc}\right)/\left(D_{pc}-D_{nc}\right)\right)\times 100$$

where D_s_, D_nc_, and D_pc_ are the absorbance of the sample, the negative control and the positive control, respectively. The experiments were run in triplicate and repeated twice.

#### Cell culture

Human renal cells (293Q cells) were obtained from the American Type Culture Collection (ATCC, No. CRL-1573). Cells were grown in Minimum Essential Medium (MEM) with 5% FBS, 2 mM glutamine (GIBCO), 0.1 mM non-essential amino acids (GIBCO) and 1 mM Sodium pyruvate (GIBCO) at 5% CO_2_ and 37 C.

#### Cell viability

Cell viability and cell proliferation were determined using a MTT (methyl tetrazolium, Sigma Aldrich, USA) assay [39]. Briefly, 293Q cells were seeded for cell viability into a 96-well plate (10,000/well) and incubated for 24 h at 37 C and 5% CO_2_. The culture medium was replaced by a fresh one supplemented with different concentrations of AmB and its analogs and incubated for 24 h. Cells were treated with AmB and its analogs (**A1** to **A21**) at concentrations of 0.01, 0.1, 1, 10, 100 and 1000 μM (AmB and **A1–A7** derivatives were dissolved in 1% v/v DMSO and **A21** in a PBS solution with pH 7.4). We did control experiments with the 1% v/v DMSO solution and found the toxicity to be 2.28% for kidney cells. This value was subtracted from all the results. We also performed a second set at concentrations of 0.1, 0.2, 0.4, 0.6, 0.8 1.0 and 10 μM (AmB and **A21** were prepared as mentioned above), in a zoom-in experiment. After treatment (24 h) the medium was gently removed and replaced with 20 μl MTT (5 mg/ml) and 150 μl of non-phenol-red medium, and incubated for 4 h. Medium from each well was discarded, followed by the addition of 200 μl DMSO and 25 μl Sorensen’s glycine buffer (glycine 0.1 M, NaCl 0.1 M, pH 10.5) to each well. When the formazan crystals were dissolved, the optical density was determined on a microplate reader (Bio-Rad) at a 590 nm wavelength. Untreated cells served as non-treatment control cell viability. The results represented a percentage of the relative viability of cells in comparison to the untreated control. MTT results are presented as measurements relative to control values, expressed as percentages.

#### Animals

Male adult Balb-c mice (Harlan Laboratories Inc. Mexico) were used. The animals were housed in a temperature and humidity controlled environment and were allowed food (Standard Purina Chow Diet, Mexico) and water ad libitum. All procedures were approved by the Institutional Animal Care and Use Committee of the Veterinary Medical School at the Universidad Nacional Autónoma de México. Experiments were conducted following the rules and principles set in the Guide for the Care and Use of Laboratory Animals (Ref: Revised guide for the care and use of laboratory animals. NIH guide. 1996; 25 [40]).

**Disseminated candidiasis in adult mice:** A *C*. *albicans* blastospore cell suspension of a chosen strain at desired concentration in PBS was prepared. Inoculum concentrations were of 10^5^ viable blastospores per mouse. *C*. *albicans* (ATCC 10–231) was obtained from ATCC and was subcultured on Yeast-peptone-dextrose (YPD), agar/broth was used for growing the strain 24 h prior to infection. The inoculum was prepared by placing three to five colonies in 5 ml of sterile 0.15 M NaCl warmed to 35 C. Fungal counts of the inoculum, determined by viable counts on SDA, were 6 ± 0.2 log_10_ CFU/ml. Disseminated infection was produced by injecting the inoculum via the dorsal tail vein using a 1ml tuberculin syringe and a 27-G, 1/2-in. needle. Standard injection volumes ranged from 100 to 200 μl. Infected animal were followed for a period of 21 days. Mice were observed daily for disease symptoms. These included weight loss, increased/decreased movement, abnormal posture (e.g., hunched back), and trembling. After this period, animals were treated with the antifungal treatments.

**Pharmacological treatments and sample collection:** Mice were randomly distributed into the following groups of n = 6:

1. Control mice received 0.3 ml of PBS by IP, three times per week
2. Candidiasis
3. Candidiasis + AmB (DMSO), 4 mg/kg IP, every day per 15 days
4. Candidiasis + AmB (Abelcet^®^), 4 mg/kg IP, every day per 15 days
5. Candidiasis + **A21**, 4 mg/kg IP, every day per 15 days
6. Candidiasis + **A21**, 12 mg/kg IP, every day per 15 days

Drugs were administered via IP in a single dose and no more than 300 μl. AmB (DMSO) was prepared in a 1% v/v DMSO solution. The maximum tolerated dose of DMSO for mouse is 2.5 mg/kg/day [41], hence the amount of DMSO applied is innocuous. Abelcet^®^ was prepared with water for injection USP and **A21** was dissolved in a PBS solution with pH 7.4.

**Organ harvest and fungal burden determination:** The kidneys, lungs, intestine, liver and blood of each mouse were immediately removed and were placed in sterile 0.15 M NaCl at 4 C. The organs were homogenized and were serially diluted 1:10. Aliquots were plated onto YPD for viable fungal colony counts after incubation for at 35 C for 24 and 48 hr. The lower limit of detection was 100 CFU/g of tissue. The number of colonies were counted and calculated and the results were expressed as CFU/g of tissue. Six mice were used to compute average and standard deviations. Tissue fragments were fixed in 4% formaldehyde solution, dissolved in phosphate-saline buffer (pH 7.4), dehydrated in alcohol, and embedded in paraffin. Four-micrometer paraffin sections were stained with hematoxylin and eosin (H&E) and subjected to histopathological examination.

**Acute toxicity:** Two hundred and sixty Balb-C mice were used and divided randomly into treatment groups of 10 animals each (five females and five males). Animals were obtained from random breeding in a closed colony. The control group received the vehicle (0.3 ml sodium deoxicholate/phosphate saline buffer [pH 7.4]). The use of deoxicholate/phosphate was in order to check the toxicity of the normal clinical formulation of AmB and since the LD_50_ is 36 mg/kg [42] we expected to be innocuous as it was found. Five others groups were treated with AmB (DMSO), at concentrations of 22, 25, 28, 30 and 35 mg/kg. Ten other groups were treated with lipid complex AmB (Abelcet^®^) at concentrations of 22, 25, 28, 30, 35, 40, 50, 100, 200 and 300 mg/kg, and ten others with **A21** at 22, 25, 28, 30, 35, 40, 50, 100, 200 and 300 mg/kg. AmB (DMSO), lipid complex AmB and **A21** were administered via IP in a single dose to mice once they had fasted for 18 h. AmB was prepared with 1% v/v DMSO, Abelcet^®^ was prepared with water for injection USP and **A21** was dissolved in a PBS solution with pH 7.4. Mortality and clinical signs (general appearance, posture/body position, consciousness/attitude, behavior, breathing, and salivation/vomiting) were recorded at 0.5, 1, 2, 4, 8, 12 h and 24 h after injection. Those animals that died during the observation period, as well as rodents that survived to the end of the observation period, were autopsied. The concentrations in which 50% mortality (LD_50_) occurred were obtained graphically by probit analysis, plotting concentration against mouse mortality [43]. After 24 h, all data were summarized in tabular form, showing for each test group the number of animals used, the number of animals displaying signs of toxicity and the number of animals found dead during the test, time of death of individual animals, a description and the time course of toxic effects and reversibility, and necropsy findings. 51 animals died in the course of these experiments and the determined cause of death was nephrotoxicity and hepatotoxicity.

**Animal sacrifice:** All animals that survived after study were killed for humane reasons and 200 mg/ml sodium pentobarbitone at a dosage of 200 mg/kg was used.

**Histopathological analysis:** Tissue fragments of treated and control animals were fixed in 10% formaldehyde solution, dissolved in phosphate-saline buffer (pH 7.4), dehydrated in alcohol and embedded in paraffin. Four-micrometer paraffin sections were stained with hematoxylin and eosin (H&E) and subjected to histopathological examination.

### Molecular Dynamics

We performed Molecular Dynamics studies of the AmB and the *L*-Histidine derivative of AmB in aqueous solutions using the GROMOS 53A6 set of parameters [44] and ~7000 SPC water molecules. In order to study the drug dimerization process we implemented the umbrella sampling technique [45].

All MD simulations were performed with Gromacs 4.5 [46] at 1 atm and 300 K. Both the temperature and pressure were maintained close to their target values using the Berendsen [47] weak coupling algorithm. A twin-range cut-off (0.8–1.4 nm) was used for the non-bonded interactions. Interactions within the short-range cutoff were evaluated every time step (2 fs), whereas interactions within the long-range cutoff were evaluated every 10 steps together with the pair-list. To correct for the truncation of electrostatic interactions beyond the long-range cutoff, the Reaction-Field correction [48] was applied (ε = 78). Bond lengths were constrained using the LINCS [49] algorithm for AmB and **A21**, and the SETTLE [50] algorithm for the water. After energy minimization a 50 ps simulation was performed with position restraints applied on all heavy atoms of the AmB or **A21** molecules. The systems were then equilibrated for 5 ns without structural restraints. MD trajectory production and analysis was performed after this point.

The relative orientation of each dimer pair was controlled by the use of six soft harmonic potential restraints, technique known as the virtual bond algorithm [51]. This simplifies the task of sampling a high number of degrees of freedom. The relative interaction strength of the interfaces can be compared between all cases since the imposed restraints were included in the unbiased procedure [52], which is a six-dimensional extension of the weighted histogram analysis method (WHAM) [53]. The comparison of the PMF profiles assumes that the relative orientation of the two monomers does not matter at long distances, i. e., the system is insensitive to the relative orientation of the monomers at the larger distances considered, typically when the distance between the center of mass (COM) of each monomer is greater than 10 Å.

#### Potential of mean force (PMF)

The PMF computation was obtained as a function of a reaction coordinate, ξ, defined as the distance between the center of mass of the monomers. The dimerization free energy is then computed as:

$$\Delta G=-k_{B}T\text{ln}K,$$

where the equilibrium constant

$$K=\int_{b}\rho \left(\xi \right)d\xi /\int_{u}\rho \left(\xi \right)d\xi ,$$

and

$$\rho \left(\xi \right)=2\pi \xi \text{exp}\left(-PMF\left(\xi \right)/k_{B}T\right),$$

Where *k*_*B*_ is the Boltzman constant, *T* is the temperature, and *ξ* is the reaction coordinate. The *b* subscript denotes the bound state, which includes only the first, most-pronounced minimum in the PMF profile, and *u* refers to the unbounded state. The prior concepts and methodology were used by Neumann et al. [54] for calculating the extent of the antibiotic dimerization. The sampling of the reaction coordinate was done by dividing the studied interval (4–25 Å) into equally sized windows, applying biasing forces on the six parameters used by the VBA method to describe the system in order to overcome free energy barriers. For each of these windows, 50 ns independent simulations were generated, sufficiently longer than the characteristic time of the drugs internal dynamic processes, which may affect the dimerization equilibrium. The starting structure for each window was obtained by a steered MD simulation, in which the drug molecules forming a dimer in a particular conformation were pulled away from each other with a constant velocity, up to the final dissociated distance. All systems in each window were allowed to relax, with the distance between monomers harmonically restrained. The errors were obtained using a bootstrap procedure in which the trajectories were cut in blocks of one fourth of the total length. 25 bootstraps ensembles were generated to calculate the average and errors.

#### Expected drug dimerization

For a moderately large number of molecules, at low concentration, the probability of finding *m* dimers obeys a Poisson distribution,

$$P_{m}=\lambda^{m}e^{-\lambda }/m!,$$

with

$$\lambda =KN_{A}N_{B}\left(v/v_{0}\right).$$

Where *K* was defined in the description of the PMF computation, *N*_*i*_ is the number of molecules of species *i*, *v* is the total volume of the system and *v*_*0*_ is a standard volume of normalization (1.66 nm3), (see Ref [55]).

#### Molecular models of AmB and A21

The models we use are based on the GROMOS 53A6 set of parameters [42,56,57]. The 53A6 set of parameters was developed to include parameterization of partitioning free energy data, in particular solvation of molecular building blocks in water and alkanes, respectively. In comparison to earlier GROMOS force fields, changes are especially found in non-bonded parameters, both Lennard-Jones parameters and partial charges. Aromatic groups are described in more detail; instead of a united atom force field for aromatic groups, the H-atoms on aromatic rings are described explicitly to account more realistically for the charge distribution. The ester group is part of the standard building blocks in 53A6 [44]. Parameters for the functional groups are derived by fitting to experimental data for small molecules representative of the functional group, e.g. ethanol, propanol, and butanol for the alcohol moieties. GROMOS87 uses the density and heat of vaporization of small molecules for parameterization of non-bonded interactions. These are the popular Lennard-Jones 6–12 potential (LJ), and the electrostatic potential using Coulomb’s law. Both potentials are used with a cut-off: pair interactions at distances larger than 1.4 nm are ignored. For the LJ interaction, straight cut-off is used; the potential discontinuously drops to zero at the cut-off. The Coulomb interaction is modified by the so-called reaction field scheme due to Tironi et al. [58], which models screening of the charge-charge interactions due to a surrounding medium and in practice modifies the electrostatic interaction so that it smoothly goes to zero at the cut-off. Parameters for bonded interactions, bond stretching, angle bending and torsional motions are largely taken from spectroscopic (X-ray) data.

Atom types and their corresponding charges for AmB and **A21** molecular models are shown in Table 2. Based on values defined for a specific atom type in the GROMOS 53A6 force field, partial charges were slightly modified in order to account for the corresponding chemical environment (2% in average, 14% in the worst case), and to balance each charged chemical group in the molecule (zero net charge). The AmB molecule, and similarly the **A21** molecule, was thought to consisting of: a sugar molecule substituted with an ammonium group, which we will call the “head”, linked to a macrocycle containing part of a sugar-like moiety substituted by a carboxylate ion, a polyol, an ester, and a polyene, which we will call the “tail”, with the same molecular characteristics. The choices for parameters for the alkyl backbone (alanine, valine, etc.), polyols (analogous to serine and tyrosine), ammonium moiety (lysine), and carboxylate moiety (glutamic and aspartic acid) are straightforward. Sugars required some modifications to the standard alkyl and alcohol parameters in conjunction with the ring geometry. Parameters consistent with GROMOS87 are described in a number of publications. We chose those employed in a study of simple glycolipids (glucose with a single alkyl tail) as described by van Buuren et al. [59], which would be appropriate for the study of AmB and **A21** interaction with lipid bilayers. GROMOS87 also describes some co- factors for proteins: the polyene moiety was modeled analogous to the building block retinol. This leaves the ester moiety as the only non-standard building block in AmB. Breaking this group in pieces, the C = O group mostly resembles the C = O group in the amide backbone building block. For the ester O and connected methylene, we chose the same atom types as the ether O and connected CH1 atoms in sugars. Bond, angle parameters and charges were then taken from what in our view is the best available set compatible with GROMOS87; the parameters used by to Chiu et al. [60] for phospholipids.

**Table 2:** Force field definition for AmB and A21 atoms. Values for atom type, atom name, partial charge, and mass are shown. In the case of **A21**, charges of the R chemical group were taken from the ARGN [44] or HISA [57] 53A6 GROMOS residues. Atom name labels are mapped to the corresponding structure, shown in Fig 6.

AmB				
nr	type	atom	charge	mass
1	OA	O15	-642	15.9994
2	H	HAA	410	1.0080
3	CH1	C38	232	13.0190
4	CH1	C39	127	13.0190
5	NL	N1	129	14.0067
6	H	HAK	248	1.0080
7	H	HAL	248	1.0080
8	H	HAJ	248	1.0080
9	CH1	C40	232	13.0190
10	OA	O16	-642	15.9994
11	H	HAZ	410	1.0080
12	CH1	C41	210	13.0190
13	CH3	C42	0	15.0350
14	OA	O17	-420	15.9994
15	CH1	C37	525	13.0190
16	OA	O14	-525	15.9994
17	CH1	C11	210	13.0190
18	CH2	C10	0	14.0270
19	CR1	C13	0	13.0190
20	CR1	C14	0	13.0190
21	CR1	C15	0	13.0190
22	CR1	C16	0	13.0190
23	CR1	C17	0	13.0190
24	CR1	C18	0	13.0190
25	CR1	C19	0	13.0190
26	CR1	C20	0	13.0190
27	CR1	C21	0	13.0190
28	CR1	C22	0	13.0190
29	CR1	C24	0	13.0190
30	CR1	C25	0	13.0190
31	CR1	C26	0	13.0190
32	CR1	C27	0	13.0190
33	CH1	C28	0	13.0190
34	CH3	C35	0	15.0350
35	CH1	C29	232	13.0190
36	OA	O11	-642	15.9994
37	H	HAB	410	1.0080
38	CH1	C30	0	13.0190
39	CH3	C33	0	15.0350
40	CH1	C31	160	13.0190
41	CH3	C32	0	15.0350
42	OE	O4	-360	15.9994
43	C	C1	580	12.0110
44	O	O9	-380	15.9994
45	CH2	C12	0	14.0270
46	CH1	C23	232	13.0190
47	OA	O10	-642	15.9994
48	H	HAC	410	1.0080
49	CH2	C34	0	14.0270
50	CH1	C45	232	13.0190
51	OA	O19	-642	15.9994
52	H	HAD	410	1.0080
53	CH2	C58	0	14.0270
54	CH2	C59	0	14.0270
55	CH1	C60	232	13.0190
56	OA	O21	-642	15.9994
57	H	HAE	410	1.0080
58	CH1	C61	232	13.0190
59	OA	O22	-642	15.9994
60	H	HAF	410	1.0080
61	CH2	C2	0	14.0270
62	CH1	C3	232	13.0190
63	OA	O5	-642	15.9994
64	H	HAG	410	1.0080
65	CH2	C4	0	14.0270
66	CH0	C5	337	12.0110
67	OA	O6	-642	15.9994
68	H	HAH	410	1.0080
69	CH2	C6	0	14.0270
70	OA	O7	-315	15.9994
71	CH1	C9	210	13.0190
72	CH1	C8	0	12.0110
73	C	C36	270	12.0110
74	OM	O12	-635	15.9994
75	OM	O13	-635	15.9994
76	CH1	C7	232	13.0190
77	OA	O8	-642	15.9994
78	H	HAI	410	1.0080
**A21**				
nr	type	atom	charge	mass
1	NZ	N1	-0.8800	14.0067
2	H	HAA	0.4100	1.0080
3	OA	O15	-0.6420	15.9994
4	CH1	C38	0.2320	13.0190
5	CH2	C39	0.0000	14.0270
6	CH1	C40	0.2320	13.0190
7	OA	O16	-0.6420	15.9994
8	H	HAI	0.4100	1.0080
9	CH1	C41	0.2100	13.0190
10	CH3	C42	0.0000	15.0350
11	OA	O17	-0.4200	15.9994
12	CH1	C37	0.5250	13.0190
13	OA	O14	-0.5250	15.9994
14	CH1	C11	0.2100	13.0190
15	CH2	C10	0.0000	14.0270
16	CR1	C13	0.0000	13.0190
17	CR1	C14	0.0000	13.0190
18	CR1	C15	0.0000	13.0190
19	CR1	C16	0.0000	13.0190
20	CR1	C17	0.0000	13.0190
21	CR1	C18	0.0000	13.0190
22	CR1	C19	0.0000	13.0190
23	CR1	C20	0.0000	13.0190
24	CR1	C21	0.0000	13.0190
25	CR1	C22	0.0000	13.0190
26	CR1	C24	0.0000	13.0190
27	CR1	C25	0.0000	13.0190
28	CR1	C26	0.0000	13.0190
29	CR1	C27	0.0000	13.0190
30	CH1	C28	0.0000	13.0190
31	CH3	C35	0.0000	15.0350
32	CH1	C29	0.2320	13.0190
33	OA	O11	-0.6420	15.9994
34	H	HAB	0.4100	1.0080
35	CH1	C30	0.0000	13.0190
36	CH3	C33	0.0000	15.0350
37	CH1	C31	0.1600	13.0190
38	CH3	C32	0.0000	15.0350
39	OE	O4	-0.3600	15.9994
40	C	C1	0.5800	12.0110
41	O	O9	-0.3800	15.9994
42	CH2	C12	0.0000	14.0270
43	CH1	C23	0.2320	13.0190
44	OA	O10	-0.6420	15.9994
45	H	HAC	0.4100	1.0080
46	CH2	C34	0.0000	14.0270
47	CH1	C45	0.2320	13.0190
48	OA	O20	-0.6420	15.9994
49	H	HAD	0.4100	1.0080
50	CH2	C58	0.0000	14.0270
51	CH2	C59	0.0000	14.0270
52	CH1	C60	0.2320	13.0190
53	OA	O21	-0.6420	15.9994
54	H	HAE	0.4100	1.0080
55	CH1	C61	0.2320	13.0190
56	OA	O22	-0.6420	15.9994
57	H	HAF	0.4100	1.0080
58	CH2	C2	0.0000	14.0270
59	CH1	C3	0.2320	13.0190
60	OA	O5	-0.6420	15.9994
61	H	HAG	0.4100	1.0080
62	CH2	C4	0.0000	14.0270
63	H	HAH	0.4100	1.0080
64	OA	O6	-0.6420	15.9994
65	H	HAM	0.4100	1.0080
66	OA	O8	-0.6420	15.9994
67	CH1	C7	0.2320	13.0190
68	CH2	C6	0.0000	14.0270
69	CH0	C5	0.3370	12.0110
70	OA	O7	-0.3150	15.9994
71	CH1	C9	0.2100	13.0190
72	CH1	C8	0.0000	13.0190
73	C	C36	0.4500	12.0110
74	OM	O12	-0.4500	15.9994
75	H	HAK	0.4400	1.0080
76	H	HAL	0.4400	1.0080
77	N	N2	-0.3100	14.0067
78	H	HAN	0.3100	1.0080
79	CH3	C62	0.1600	15.0350
80	OM	O18	-0.3600	15.9994
81	O	O19	-0.3800	15.9994
82	C	C63	0.5800	12.0110
83	CH1	C64	0.0000	13.0190
84	CH2	C65	0.0000	14.0270
85	C	C66	0.0000	12.0110
86	H	HAP	0.3100	1.0080
87	NR	N3	-0.0500	14.0067
88	C	C67	0.1400	13.0190
89	NR	N4	-0.5400	14.0067
90	C	C68	0.1400	13.0190

**Fig 6:**
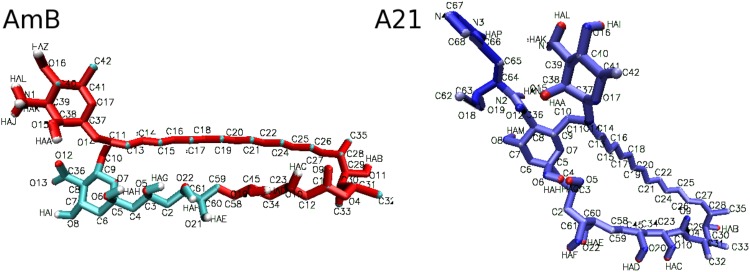
Molecular structure of AmB and A21, showing atom names according to definitions in Table 2.

**Virtual bond algorithm (VBA):** Description of the protocol used to compute the potential of mean force and the details of the VBA as biasing potential are given in [61]. In short, three anchors on each antibiotic molecule allowed for the definition of the relative orientation of the two monomers. Anchors are defined with the help of one distance, *d*, two angles, θ_1_ and θ_2_, and three dihedral angles, φ_1_, φ_2_, and φ_3_. These restraints where added to the topology of the system as harmonic restraints for distances and dihedral angles and a cosine angle potential for the regular angles. The force constants and reference values used for the restraints are given in Table 3.

**Table 3:** Virtual bond algorithm parameters used to define and control the relative orientation of the monomers for the three dimers conformations studied in this work. Angle values are given in degrees. A, B, C refer to the atom index of monomer one, and a, b, c refer to the atom index of monomer two. These sets of atoms define the anchor VBA angles used as bias potentials during the PMF calculations, with force constants k_d_, k_θ_ and k_φ_ (as in Ref [59]).

	HTA							HHA							HHP						
	[°]	A	B	C	a	b	c	[°]	A	B	C	a	b	c	[°]	A	B	C	a	b	c
θ_1_	96	30	18		120			96	30	18		149			90	30	18		108		
θ_2_	96	30			120	108		96	59			120	108		90	18			120	108	
φ_1_	0	30		18	120		108	9	30		18	108		120	0	30		18	108		120
φ_2_	-76	59		18	30		108	-96	59		18	30		120	-90	59		18	30		120
φ_3_	70	149		108	120		18	-96	149		108	120		30	90	149		108	120		30
	k_d_ = 2500 kJ mol^-1^ nm^-2^							k_θ_ = 500 kJ mol^-1^							k_φ_ = 250 kJ mol^-1^ rad^-2^						

## Results and Discussion

### Chemical synthesis

Substitutions in polyenes have been performed for a long time in the search for improved selectivity (see for example [62–65]). Here, amide substitutions were used to synthesize AmB analogues. Analogues **A1** to **A7** were synthesized from aliphatic and aromatic amines in order to increase steric and electronic effects between the amide moiety and the micosamine ring. Thus, benzyl amide was selected for preparation of **A1** in order to induce a steric effect between the aromatic ring and the mycosamine unit of the molecule. In addition, aromatic rings could favor the π-π interaction between the rings of adjacent molecules in the pore supra-structure. H-π interactions between the OH group of the micosamine and aromatic rings of **A1** could lead to steric effects affecting pore formation. Cyclohexyl amine and diisopropyl amine were selected for the synthesis of **A2** and **A3** with the purpose of comparing moderate and strong steric factors. In addition to the π-π and H-π interactions described for **A1, A4** and **A5** have the effect of a chiral carbon on the amide moiety. **A6** and **A7** were designed with the purpose of having analogues displaying UV fluorescence, to facilitate visualization on the formation of channels, while the additional presence of the heterocyclic idole ring, the methylene group and the ester functionality would lead to larger steric effects. **A6** and **A7** showed a better performance in the selectivity of fungal vs mammal cells. We thought that the presence of nitrogen groups could be partially responsible for this. We thus decided to consider a derivative with increased number of nitrogen atoms in the ring. Although histamine substitution was thought of as a possibility, we found that such a derivative reverted to the parent molecule in tissue culture tests. Therefore, we ended up with a derivative having the imidazol ring and the methyl ester to prevent hydrolysis, that is, **A21**. Post-purification yields, representative Infrared signals and High Resolution Mass Spectrometry of several examples are shown in Table 1.

### Electrophysiological experiments

These analogs were tested for transmembrane transport in POPC/cholesterol lipid bilayers, by means of the tip dip technique, in order to determine their ability to produce K^+^ leakage. Table 4 shows the open probability, i.e., the percentage time that a channel appears conducting current through the K^+^ conducting pores. It must be noted that the concentration used for all derivatives is 200 μM, whereas it is only 10 μM for AmB. This is due to the reduced formation of pores shown by most of the derivatives.

**Table 4:** Probability of opening of the different types of channels observed for: AmB and the compounds presented in Table 1 in a lecithin membrane with 30% Mol cholesterol.

	Open probability								
[] μM	200	200	200	200	200	200	200	200	10
Type ^a^	A1	A2	A3	A4	A5	A6	A7	A21	AmB ^b^
**I**	2%	5%	< 1%	7%	10%	23%	14%	n.o.	0.59%
**II**	1%	< 1%	n.o.	3%	2%	2%	7%	13.7%	0.26%
**III**	< 1%	< 1%	n.o.	1%	< 1%	< 1%	< 1%	n.o.	0.08%
**IV**	< 1%	< 1%	n.o.	< 1%	< 1%	< 1%	< 1%	n.o.	4.06%
**V**	< 1%	< 1%	n.o.	< 1%	< 1%	< 1%	< 1%	n.o.	1.81%
**VI**	< 1%	< 1%	n.o.	< 1%	< 1%	n.o.	n.o.	n.o.	0.93%

^a^ Ref [12]

^b^ Ref [14]

n.o. = not observed

Furthermore all compounds, except for **A3** and **A21**, exhibit the first five channel types, with the larger conductance channel (~ 70 pS) appearing in half the compounds. Of course, the different concentrations required for channel expression could lead to different aggregation of the compounds in an aqueous solution: either the derivatives require a large concentration to aggregate or larger aggregates are needed for insertion of the derivatives into the membrane. However, the fact that the smallest conductance channel (~ 4 pS) is by far the most frequent also indicates that the expression of the derivatives’ channels is also hindered.

### Pharmacological experiments

Reduced activity in cholesterol-containing membranes could perhaps be reflected in increased selectivity. Hence, the derivatives were tested in pharmacological studies, of fungal cells, erythrocytes and kidney cells; the results are presented in Table 5. The pharmacological results agree with the electrophysiological experiments: there is very poor toxicity toward mammalian cells. Given that there is also poor toxicity towards fungal cells in most of the derivatives, no major advantage could be obtained, except in the cases of **A7** and **A21**.

**Table 5:** Pharmacological tests of compounds of Table 1.

Compounds	*Candida albicans* ATCC 752	*Candida albicans* ATCC 10231	*Candida krusei ATCC 6258*	Hemolysis in human red blood cells	Cytotoxic activity on kidney cells ATCC CRL-1573
	IC_50_[μM]	IC_50_[μM]	IC_50_[μM]	HeC_50_ [μM]	TC_50_ [μM]
**AmB**	1	0.20	>10	7	9.7
**A1**	>100	>100	>100	52	100
**A2**	>100	>50	>100	45	120
**A3**	>100	>50	>100	53	180
**A4**	>100	>50	>100	46	80
**A5**	>100	>50	>100	42	100
**A6**	>100	>50	>100	58	120
**A7**	8.2	1.5	>10	80	220
**A21**	0.67	0.28	7.5	409	>500

In the case of **A7** there is a reduction in toxicity that is larger for the mammalian cells than the fungal ones, leading to an increase in selectivity, albeit a reduced one. These results and the idea of promoting interaction with the membrane led to using *(L)*-histamine as a substitute, which produced a derivative with increased selectivity. Nonetheless, this amide reverted to AmB action on kidney cells after 24 h, probably due to the action of proteases. In order to prevent this possible hydrolytic pathway, we screened a series of AmB amide analogues containing nitrogen heterocyclic ring systems where the *(L)*-histidine methyl esther **21** led to the analogue known as **A21**. This produced the best results.

As may be seen in Table 5, the performance of this derivative exhibits a considerable increase in selectivity. This derivate is presented in Fig 2.

### Electrophysiological experiments

We took a more detail look at the derivative’s electrophysiology and found that it exhibits a pattern for total average conductance (the average conductance when all types of channels are considered) in ergosterol- and cholesterol- containing membranes, with much increased selectivity. These results are presented in Table 6, where they are compared to those of AmB. Selectivity is defined per the following formula:

$$Selectivity=\frac{G_{ergosterol}}{G_{cholesterol}}\frac{log{\left[polyene\right]}_{cholesterol}}{log{\left[polyene\right]}_{ergosterol}}$$

Where G_ergosterol_ refers to the conductance in ergosterol-containing membrane, G_cholesterol_ refers to the conductance in cholesterol-containing membrane, [polyene]_cholesterol_ refers to the corresponding concentration of the polyene in the cholesterol-containing membrane and [polyene]_ergosterol_ refers to the corresponding concentration of the polyene in the ergosterol-containing membrane. The increment in selectivity (4.33 fold) of compound **A21** is in agreement with the pharmacological results presented in Table 5.

**Table 6:** Average total conductance produced by AmB and derivative A21 in POPC membranes with 30% Mol cholesterol or ergosterol; selectivity is described in the text.

Compound	Concentration (μM)	Average conductance (fS) cholesterol	Average conductance (fS) ergosterol	Selectivity	Selectivity increment
**AmB**	15	23 ± 15	---	3.5	1
	5	---	48 ± 11		
**A21**	200	108 ± 11	---	15.16	4.33
	10	---	712 ± 33		

**Fig 7:**
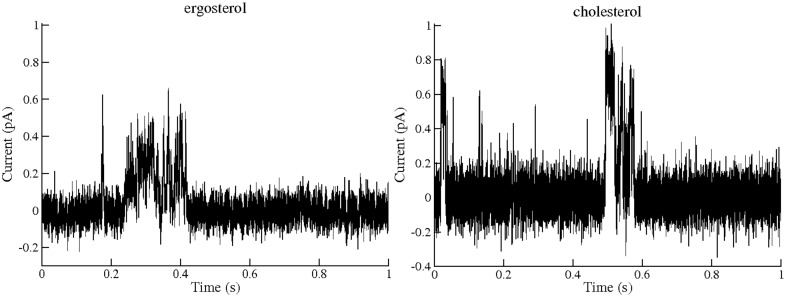
Example of single channel along 1 s activity of Amphotericin B in a POPC membrane containing 30% Mol ergosterol or 30% Mol cholesterol, 25°C, 100 mV and concentrations of 5 and 15 μMol respectively. These records were treated as described in the Methods section.

**Fig 8:**
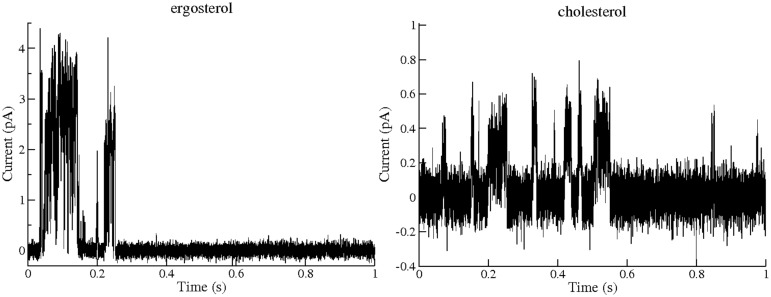
Example of single channel along 1 s activity of A21 in a POPC membrane containing 30% Mol ergosterol or 30% Mol cholesterol, 25°C, 100 mV and concentrations of 10 and 200 μMol, respectively. A larger channel for the ergosterol-containing membrane is presented (note the different scale) since, in this case, it is very common. These records were treated as described in the Methods section.

Furthermore, the single channel experiments allowed us to compare the transmembrane pores formed by AmB and **A21** that enable the passage of K^+^. Figs 7 and 8 show the corresponding single channel currents for AmB and the **A21** derivate; smaller channels (type I) are common for AmB and **A21** in cholesterol-containing POPC membranes. In ergosterol-containing POPC membranes, **A21** presents a channel of 6 pS as the most common one, similar to type IV of Ref [13].

### Spectroscopic experiments

**Fig 9:**
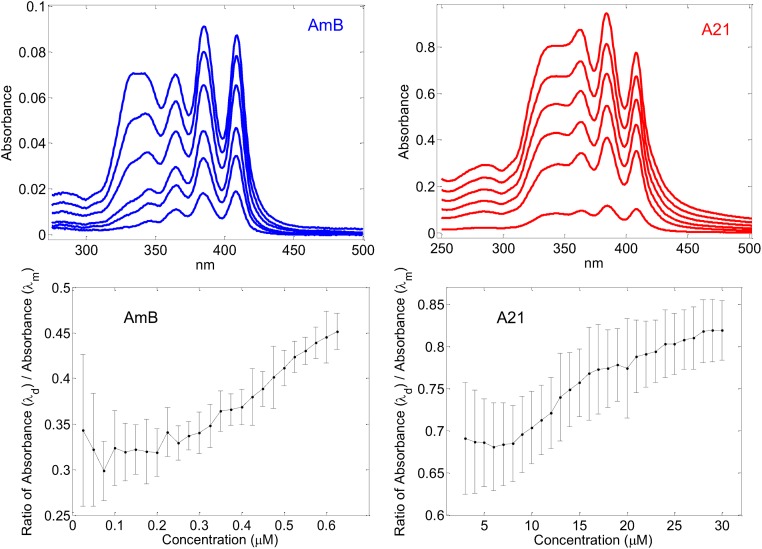
Top figures: Absorption spectra in a PBS solution at 30 C of different concentrations of the polyenes. The left hand side presents AmB spectra at concentrations of 0.15, 0.3, 0.6, 0.9, 1.2 and 1.5 μMol, corresponding to a small- to- large profile. The right hand side presents **A21** spectra at concentrations of 2, 8, 14, 20, 26, and 32 μMol, corresponding to a small- to large- profile. Bottom figures: Ratio between absorbance at 409 nm and that at 347 nm as a function of concentration. Left hand side, AmB; and right hand side, **A21**.

An additional advantage of the **A21** derivate is its increased solubility in water; **A21** readily dissolves at 50 mMol, whereas AmB does so only in the μMol range. Also, the dimerization of both components seems to occur at different concentrations in aqueous solution. The profile of UV Absorbance of AmB has been observed to be different in its monomeric or aggregated form [66]. An estimate of polyene aggregation can be obtained from the ratio between the Absorbance at a wave length of 409 nm and the corresponding one at a wave length of 347 nm. When plotted as a function of the concentration, it is possible to determine the threshold for the onset of dimerization. The first wave length is characteristic of a monomeric state of the polyene, whereas aggregation produces absorbance at the second wave length [56,66]. Care should be taken to determine these spectra in inert atmosphere since it has been shown that oxidation of AmB gives rise to bands in the same region that the band used for determining dimerization [67]. The spectra corresponding to the **A21** derivative at different concentrations are presented in Fig 9, as well as the corresponding spectra of AmB. As may be seen the range of concentrations at which the profiles change are quite distinct. Fig 9 also shows the profile of the rate of Absorbance at 347 nm / Absorbance at 409 nm of derivative **A21** and AmB. This ratio is constant at first, reflecting the presence of a single profile. At certain concentration, the rate starts to increase as aggregation begins to appear. A bilinear adjustment to this profile shows a dimerization onset for **A21** at 8 μMol whereas this onset for AmB occurs at 0.2 μMol. The latter is smaller than the previously reported dimerization at 1 μg/ml [66], that is ~ 1 μMol, probably due to the effect of avoiding oxidation. The increased solubility of **A21** yields an advantage in the therapeutic use of the derivative, and its increased selectivity could be a result of the difference in the onset of dimerization. See the Methods section for the details of the Absorbance experiments.

### Molecular dynamics

**Fig 10:**
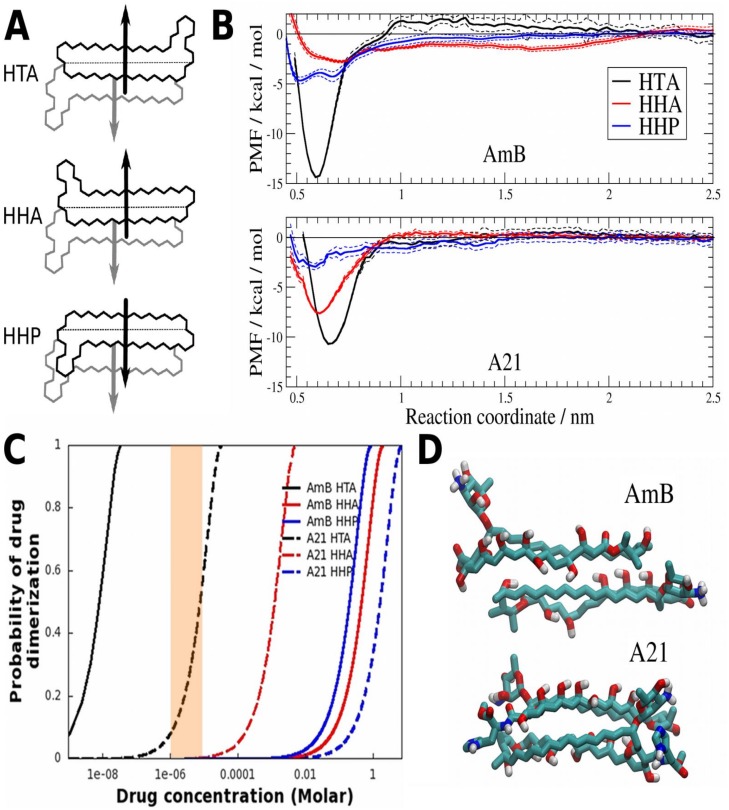
Thermodynamic molecular characterization of drug dimer formation. A) Schematic representation of the three conformations of dimers studied. From the top, head-to-tail with anti-parallel dipole moment (HTA), head-to-head with anti-parallel dipole moment (HHA), and head-to-head with parallel dipole moment (HHP). B) Potential of mean force (PMF) as a function of reaction coordinate ξ in the HTA (black), HHA (red) or HHP (blue) configurations, for AmB (top) and **A21** (bottom). Estimated error shown with dashed lines. C) Expected drug dimerization as a function of molar concentration, for AmB (solid lines) and **A21** (dashed lines) on HTA (black), HHA (red) or HHP (blue) configurations. D) Molecular dimer structure in equilibrium in the HTA configurations for AmB (top) and **A21** (bottom). Inkscape, xmgrace and VMD were used to make panels A, B and C, respectively.

We also looked into the molecular basis of the increased selectivity of **A21** from a theoretical standpoint, using a molecular modeling and a thermodynamics approach. Aggregation is one of the proposed molecular factors responsible for the mechanism behind of AmB selectivity. Given the previous results and following this hypothesis, we carried out a comparative study of the dimeric aggregation of AmB vs that of **A21** in an aqueous solution. Long-time-scale simulations were performed on two equivalent systems. We placed two molecules of either AmB or **A21** in a water solution having 150 mM NaCl, a temperature of 27 C and a volume large enough to have low concentrations of the drug. Fig 10 summarizes the results.

The dipole moment predicted by our force field yielded a value of 35 D for the AmB molecule. In contrast, the **A21** molecule has an electrical dipole of 10 D. These values do not change with the formation of a dimer since we do not include polarization effects. We speculate that the significant difference in the dipole values could explain the molecular aggregation process. Hence, different dimer configurations were considered in this work, namely, head-to-head (HH) or head-to-tail (HT), with parallel (P) or anti-parallel (A) relative orientations of the dipole moment. Schematic drawings of these arrangements are presented in Fig 10A. The molecular dynamics simulations showed stable configurations of the HTA, HHP and HHA dimers. However, the HTP configuration disassembled within a couple of nanoseconds and eventually reassembled in the HTA configuration. This effect happened for both AmB and **A21** HTP dimers. We decided to exclude the HTP configuration from further analysis due to its very unstable behavior.

To characterize the fact that the self-association thermodynamic process of the drug depends on the relative dimer orientation, we computed the potential of mean force (PMF), reported for AmB and **A21** in Fig 10B. This strategy has already been used successfully, e. g., to study protein-protein interactions inside a membrane. Details of this methodology are provided in [61] and in the Methods section. Moreover, to quantify the energetic cost involved in the monomer−monomer interactions, we calculated the dimerization free energy, ΔG, from the PMF profiles. The ΔG values for a standard state imposed by the simulation conditions are shown in Table 7. As expected, the most favorable dimerization energies correspond to a configuration with antiparallel dipoles. This result applies to both AmB and **A21** in the HTA orientation, which presents the lowest local minimum in the PMF profiles. Furthermore, AmB has an energy advantage of ~ 4 kcal/mol in comparison to **A21**. The free energy difference means that the AmB HTA dimers are more stable in an aqueous solution than the **A21** counterpart. In other words, **A21** solubility in water is greater than AmB.

**Table 7:** Free energy of dimerization of AmB and A21 in an aqueous solution in three possible configurations (HTA, HHA, HHP). The distance between the center of mass (COM) of the polyene ring of the monomers for the geometry of minimal energy is also given.

	AmB		A21	
	d_m_ [Å]	ΔG [kcal/mol]	d_m_ [Å]	ΔG [kcal/mol]
**HTA**	5.9	-11.9	6.6	-8.1
**HHA**	7.1	-1.6	6.1	-5.1
**HHP**	5.1	-2.1	5.8	-0.9

It must be mentioned that the ΔG value of the HHP configuration of AmB is found between the two antiparallel configurations. We can explain this particular behavior by considering the amphiphilic nature of the molecule. Reports indicate that the AmB molecule has a high tendency to undergo lipophilic interactions with other AmB monomers in aqueous media [57]. Starzyk et al. [56] made the first calculation to quantify the total electrostatic and van der Waals contributions to the AmB-dimerization energy when two molecules are parallel or anti-parallel to each other in an aqueous solution. Despite a robust analysis, however, they did not take the dipole moment of the molecule into account. The authors suggested a hydrophobic nature of the dimerization process because the AmB polar groups are involved in the interaction with water molecules rather than stabilizing contacts in the dimer. In the parallel orientation, the AmB-AmB stability comes from strong van der Waals interactions due to a large contact surface. On the other hand, in the antiparallel geometry, the AmB dimer is stabilized mostly by the electrostatic interaction between the hydroxyl chains and the hydroxyl tail group. Hence, the energy advantage of the HHP over the HHA configuration in the AmB dimer arises from the fact that the hydrophobic effect is stronger than the electrostatic and van der Waals-type interactions. Nevertheless, the predominant energy contribution for dimerization is the one resulting from dipole-dipole interaction. We found that antiparallel dipole configurations exhibited a greater stability in all cases. Therefore, we propose that the selectivity difference between the two drugs originated in the smaller dipole moment of **A21** relative to AmB.

We additionally estimated the extent of dimerization as a function of drug concentration using the predicted equilibrium constant, K, derived from the PMF profiles. The results are shown in Fig 10C. Also, analysis of the equilibrated molecular structure of the HTA conformation suggests that **A21** weakens dimers because the interaction between the polyene rings is perturbed by the presence of the L-Histidine group that replaces the AmB carboxyl group (Fig 10D). Overall, the predicted relative behavior between AmB and **A21** in the dimerization process shows good agreement between theory and experiment. Following Huang et al. [31], we believe there is a need of dimerization for the appearance of ion channels in cholesterol-containing membranes. In order to examine this, we looked into the pharmacological effects of both compounds as a function of concentration.

### Pharmacological experiments

**Fig 11:**
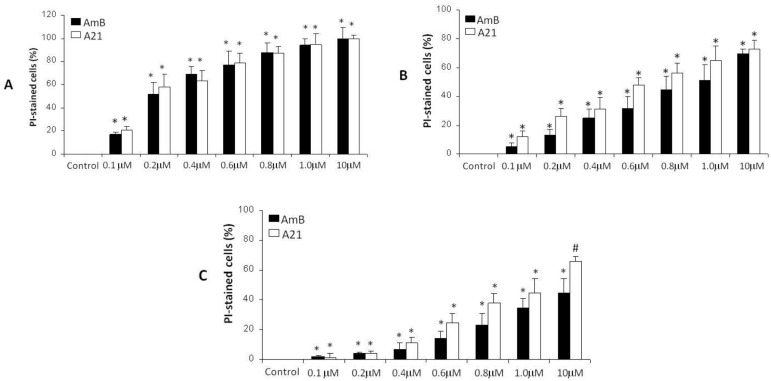
Comparative action of Amphotericin B and derivative A21 on *Candida albicans* ATCC 10231 (A), *Candida albicans* ATCC 752 (B) and *Candida krusei* ATCC 6258 (C). Cells were incubated for 24 h at 37 C under aerobic conditions, stained with propidium iodide (PI). Antifungal activity was determined by flow cytometry. The results are presented as means ± SD of 3 independent experiments. *p < 0.05 is the comparison with control value. #p< 0.05 the comparison with AmB.

The potency of the AmB and **A21** compounds against *Candida albicans* strains at different concentrations is shown in Fig 11. A very similar behavior is observed for both compounds, indicating that the antimycotic character of the parent molecule is preserved. Furthermore, **A21** presents larger toxicity in the case of *Candida krusei*, which is AmB resistant. There is a very similar trend of action of both compounds as a function of concentration of up to 10 μMol; pore expression in the ergosterol-containing membrane is therefore similar.

**Fig 12:**
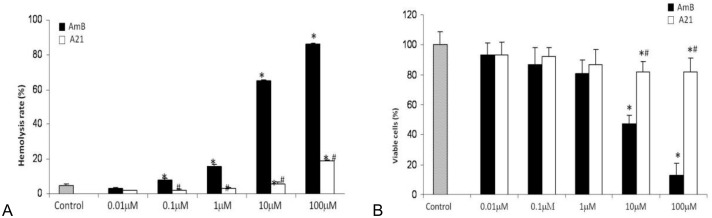
A) Comparative effect in hemolysis of human RBCs by AmB and **A21** after 2 h incubation at 37 C (data represented as mean ± SD; n = 8). Significant differences with respect to control or AmB are indicated with *p < 0.05 and #p < 0.05, respectively. B) Changes in the survival of kidney cells HEK-293Q after treatment with different concentrations of AmB (DMSO) and **A21** during a 24 h incubation period (control = 100%). The results are presented as means ± SD of 3 independent experiments. *p < 0.05 is the comparison against control value. #p < 0.05 the comparison against AmB.

Fig 12 shows a comparison of the effects that AmB and **A21** have on hemolysis at different concentrations, as well as on renal cells (293Q). **A21** has a considerably reduced hemolytic effect, which agrees with the observations of the single channel pores in cholesterol-containing membranes. The same may be seen in the case of kidney cells: at 100 μMol AmB there is almost total reduction of viability, whereas 100 μMol **A21** produces only a 10% decrease. AmB in cholesterol-containing membranes showed the effect of drug dimerization at 1 μMol and starts to affect RBC and kidney cells. On the other hand, **A21** starts dimerization around 8 μM. In Fig 12 we can observe that AmB at 1 μM has the same effect on mammal cells that **A21** at 100 μM, a difference of two orders of magnitude, close to the MD predicted difference in the dimerization onset of the compounds. On the other hand, the spectroscopic prediction on the dimerization onsets ratio for both compounds is closer to the observed ratio of the drug’s lethal toxicity, see Table 8.

**Table 8:** Comparison of the LD_50_ produced in Balb-C mice by Amphotericin B (in two presentations) and A21 derivative.

Drug	LD_50_
AmB (DMSO)	29.3 mg/kg
Abelcet^®^	> 80 mg/kg
**A21**	199.48 mg/kg

A crucial test regarding the improved safety and equivalent efficacy of the **A21** derivative needs to involve preclinical tests in mammals. This was done using groups of Balb-C mice (n = 10). After intraperitoneal (IP) injection of the compounds, most mice in the 100 to 300 mg/kg AmB groups were quiet and inactive and died approximately 12 h after the injection. The majority of mice in the 40 to 300 mg/kg AmB treatment died within 24 h following the injection. In contrast, no mice died after a treatment with 40–300 mg/kg of **A21** after 12 h, whereas most mice treated with 200 and 300 mg/kg **A21** died within 24 h following the injection. The corresponding LD_50_ obtained were 29.3 mg/kg for AmB and 199.48 mg/kg for **A21**; that is, almost a 7-fold increase in regards to AmB. This is shown in Table 8, where a comparison that includes Abelcet^®^, is presented.

**Fig 13:**
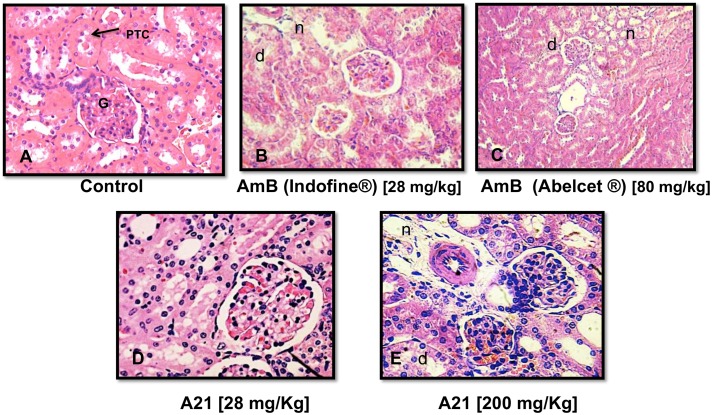
Histopathological analysis of kidney samples from animals treated, with AmB (DMSO), AmB (Abelcet^®^) and A21. (A) Appearance of normal renal tubules in a kidney from a representative uninfected male Balb-C mouse treated for 2 weeks with daily IP doses of sodium deoxycolate (control). (B) Kidney from a representative male Balb-C mouse treated with a single IP dose of 28 mg/kg AmB (DMSO), showing clusters of dilated degenerative tubules (d) containing exfoliated necrotic epithelium admixed with necrotic tubules (n). (C) Kidney from a representative male Balb-C mouse treated with a single IP dose of 80 mg/kg AmB (Abelcet^®^). (D) Kidney from a representative Balb-C mouse treated with a single IP dose of 28 mg/kg **A21** where a lesser amount of necrotic and degenerative tissue is observed. Rare tubules lined by regenerative epithelium with intraluminal protein casts are visible. (E) Kidney from a representative Balb-C mouse treated with a single IP dose of 200 mg/kg **A21**, showing clusters of dilated degenerative tubules (d) containing exfoliated necrotic epithelium, admixed with necrotic tubules (n), as well as inflammatory cells (ic). G = glomerulus; IP = intraperitoneal; PT = proximal tubule; TD = distal tubule; H&E-stained magnification 100X.

Kidney histological analysis stresses the observed difference in toxicity. Animals treated with 28 mg/kg AmB for 24 h exhibited clusters of dilated degenerative tubules, containing exfoliated necrotic epithelium and necrotic tubules, but no histological changes were observed in kidney slices from animals treated with 28 mg/kg of **A21**. However, animals treated with 200 mg/kg **A21** for 24 h showed very tight and congested glomeruli, as well as convoluted tubules with isquemic damage as shown in Fig 13, which agrees with this being the lethal dose.

In order to verify the efficacy of the **A21** compound on a candidiasis infection, produced by *Candida albicans* (ATCC 10231), two forms of AmB, a soluble one (DMSO) and a lipid complex one (Abelcet^®^), were compared to **A21**. Balb-c mice were infected and allowed to incubate for three weeks. At the end of this period, different mice groups were treated for another two weeks with the different compounds. For the three compounds, an IP dose of 4 mg/kg/day was used. This dose is higher than the therapeutic IV dose of 1 mg/kg/day. The reason for using this dose is that 5–20 mg/kg/day of AmB have been used previously when administered intraperitoneally [27]. We also considered 12 mg/kg/day for **A21**, since the augmented safety could allow a dose increase if needed. We used the 4mg/kg dose of Albecet because we found it to be as effective as the solution formulation of AmB, and we wanted to determine its toxicity at this dose vs those of AmB and **A21** at the same value that was previously used for IP application [27]. The results are summarized in Tables 9 and 10.

**Table 9:** Histopathological evaluation of infected mice treated with AmB or A21.

	Number of mice with acute kidney tubular necrosis anddegeneration vs. mice in treatment group.	Number of mice with histiocytic,neutrophilic, multifocalbronchopneumonia in lungs vs. mice in treatment group.	Number of mice with necrotic enteritis accompanied by loss of microvilli, inflammation and cellular lysis vs. mice in treatment group.	Number of mice with liver necrosis and degeneration and cholestatic liver vs. mice in treatment group.
**Candidiasis**	6/6	6/6	6/6	6/6
**AmB (DMSO) (4 mg/kg)**	2/6^a^	2/6^a^	2/6^a^	2/6^a^
**AmB(Abelcet**^**®**^**) (4 mg/kg)**	0/6^a^	1/6^a^^b^	0/6^a^^b^	1/6^a^^b^
**A21 (4 mg/kg)**	0/6^a^^b^	0/6^a^^b^^c^	1/6^a^^b^	0/6^a^^b^
**A21 (12 mg/kg)**	0/6^a^^b^	0/6^a^^b^^c^	1/6^a^^b^	0/6^a^^b^

^a^ p < 0.05 as compared with Candidiasis group;

^b^ p < 0.05 as compared with AmB (DMSO);

^c^ p < 0.05 as compared wih AmB (Abelcet^®^);

A two-tailed Fisher’s exact test was used.

**Table 10:** CFU from *C*. *albicans* recovered from intestine, liver, kidney and lung homogenates of infected mice treated with AmB and A21.

Candidiasis	Intestine*20 ± 3	Liver*30 ± 5	Kidney*18 ± 4	Lung*9 ± 3
**AmB (DMSO) (4 mg/kg)**	6 ± 2^a^	5 ± 1^a^	4 ± 1^a^	3 ± 0.5^a^
**AmB Abelcet**^**®**^ **(4 mg/kg)**	4 ± 1^a^	6 ± 1^a^	2 ± 0.3^a^	3 ± 0.1^a^
**A21 (4 mg/kg)**	10 ± 3^a^	6 ± 2^a^	2 ± 0.2^a^^b^	4 ± 1^a^
**A21 (12 mg/kg)**	6 ± 1.5^a^^c^	3 ± 0.5^a^^b^^c^	3 ± 0.1^a^^c^	2 ± 0.2^a^^b^^c^

* Log10 CFU/g of tissue.

^a^ p< 0.05 as compared with candidiasis group.

^b^ p< 0.05 as compared with AmB DMSO.

^c^ p< 0.05 as compared with AmB Abelcet^®^.

A two-tailed Fisher’s exact test was used.

Table 10 shows that all compounds alleviate the infection in a similar manner, with a slight advantage observed in the case of Abelcet^®^ and **A21**. Increasing the dose of **A21** did not remove the minimal intestinal damage that appeared with the 4 mg/kg dose. The microbiological studies of intestine, blood, kidney, and lung of infected and treated mice, show a strong infection of all organs in infected mice, whereas the organs of those treated with the two forms of AmB and the two doses of **A21** present only traces of *Candida*. In short, **A21** performs as effectively as AmB.

**Fig 14:**
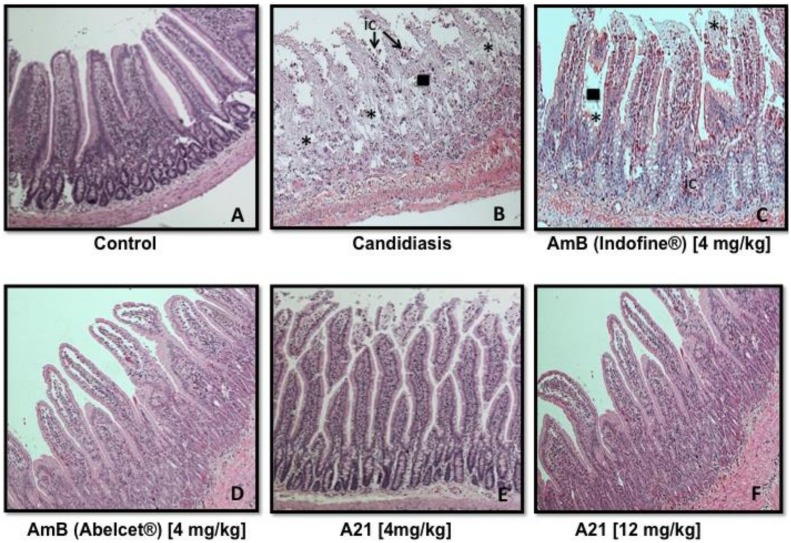
Histopathological analysis of intestinal epithelium obtained five weeks after *Candida albicans* inoculation in mice. (A) Intestinal epithelium of Balb-C mouse with normal appearance. (B) Intestinal epithelium showing severe damage with necrotic enteritis. We can observe the loss of microvilli (■), presence of inflammatory cells (ic) and cellular lysis (*) is observed. (C) Intestinal epithelium from a representative mouse with candidiasis treated with a daily IP dose of 4 mg/kg AmB (DMSO) during the last two of the five weeks. Loss of microvilli (■), presence of inflammatory cells (ic), and cellular lysis (*) is observed. (D) Intestinal epithelium from a representative mouse with candidiasis treated with a daily IP dose of 4 mg/kg AmB (Abelcet^®^) during the last two of the five weeks. The kidney appears normal and has abundant caliciform cells. (E) Intestinal epithelium from a representative mouse with candidiasis treated with a daily IP dose of 4 mg/kg **A21** during the last two of the five weeks. Epithelium with normal appearance. (F) Intestinal epithelium from a representative mouse with candidiasis treated with a daily IP dose of 12 mg/kg **A21** during the last two of the five weeks. Epithelium with normal appearance.

**Fig 15:**
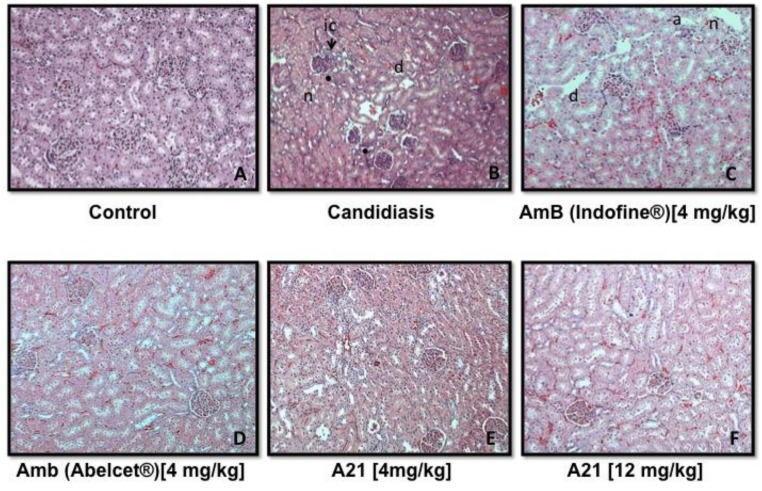
Histopathological analysis of kidney obtained five weeks after *Candida albicans* inoculation in mice. (A) Kidney of Balb-C mouse with normal appearance. (B) The kidney shows severe pyelonephritis with fungal infiltration (●). The image shows clusters of dilated degenerative tubules (d) containing exfoliated necrotic epithelium admixed with necrotic tubules (n). The inflammatory infiltration (ic) damaged the lining of the tubules. Renal tubular nephrosis was observed. (C) Kidney from a representative mouse with candidiasis treated with a daily IP dose of 4 mg/kg AmB (DMSO) during the last two of the five weeks. Clusters of dilated degenerative tubules (d) containing exfoliated necrotic epithelium admixed with necrotic (n) and apoptotic tubules (a) appear. (D) Kidney from a representative mouse with candidiasis treated with a daily IP dose of 4 mg/kg AmB (Abelcet^®^) during the last two of the five weeks, with normal appearance. (E) Kidney from a representative mouse with candidiasis treated with a daily IP dose of 4 mg/kg **A21** during the last two of the five weeks. The kidney shows normal appearance. (F) Kidney from a representative mouse with candidiasis treated with a daily IP dose of 12 mg/kg **A21** during the last two of the five weeks, showing a normal appearance.

Histological sections of the organs show, as previously observed, that **A21** caused less damage to the cells that AmB in its two presentations. Figs 14 and 15 show histological sections of the intestines and kidneys of control and treated animals.

## Conclusions

We have presented a multidisciplinary approach that advances the understanding of polyene action. Nonetheless, there are some limitations involved.

The synthesis of derivatives was made starting from an AmB compound, which is not 100% pure in as much as it also contains Amphotericin A and Amphotericin C, leading to the derivatives inheriting this impurity. However, its purity (91.78%) is similar to that of the Sigma Amphotericin B, United States Pharmacopeia (USP), Reference Standard (88.91%) as tested by HPLC. Although AmB is usually prescribed by the intravenous route, antimycotics were applied to mice intraperitoneally. As a result of this, some values may be different, such as the LD50, but the relative efficacy and toxicity between both compounds should be reliable. As previously mentioned, Molecular Dynamics results are very dependent on the force fields used, and since we had to construct the necessary force fields, they have not been extensively tested. As a matter of fact, comparison of the electrostatic description of the AmB molecule produce by this force field with that provided by an ab-initio calculation [68] shows discrepancy even in the predicted dipole moment. Nonetheless, we can expect relative comparisons between the two polyenes to be more reliable. This is supported by the agreement shown with the experimental predictions on relative dimerization. The electrophysiological comparison between Amphotericin and its derivatives was performed in very simple systems, membranes patches of POPC containing ergosterol or cholesterol in order to mimic the difference between fungal and mammal cells. The validity of the model was supported by subsequent results in microbiology and animal experiments. Thus, the multidisciplinary character of the research gives more confidence regarding the obtained results.

Our findings are based on the idea that membrane structure is behind polyene selectivity and results showed that nitrogenated amides with a reduced dimerization lead to an increase in selectivity and, therefore, to reduced collateral toxicity. We advance this hypothesis based on the proposed mechanism of action derived from channel formation, which is affected by self-association of the polyene in the aqueous solution, as proposed by Huang [31] and Starzyk [56]. We were not able to structure an explanation of the increase selectivity of derivative **A21** based on the sponge model. Our derivative modifies a region opposed to the proposed region of interaction of the polyene with the sterol in this latter model. Moreover, difference in aggregation of polyenes does not seem to play a role in this model.

Table 11 shows a summary of the distinct effects of AmB and derivate **A21** on fungal and mammalian cells, as well as a comparison with two other recent AmB derivatives with increased selectivity that have appeared in the literature. The increase in selectivity produced by all new derivatives is clear, as is the advantage of compound **A21**, encouraging its possible therapeutic use.

**Table 11:** Comparison of selectivity (fungal vs mammal cells) of AmB and several of its derivatives. MIC_50_ for *Candida* strains, hemolysis and kidney cell damage are shown. Selectivity, which is also included, is defined as the ratio of concentrations at which MIC_50_ occurs for *Candida* strains with respect to the concentration for HE_50_ or the MIC_50_ of kidney cells.

**Carreira E.** [28]				
**Compound**	**MIC**_**50**_**-*Candida albicans*DSY294 (**μ**M)**	**HE**_**50**_**-Erythrocytes (**μ**M)**	**Selectivity**	**AmB selectivity / Compound selectivity**
**AmB**	0.4	4	10	1
**Diamine 3**	0.2	10	50	5
**Diamine-ester 9**	0.25	50	200	20
**Diamine amide 10**	1.0	30	30	3
**Wilcock et al.** [21]				
**Compound**	**MIC-*C*. *albicans* (**μ**M)**	**HE**_**50**_**-Erythrocytes (**μ**M)**	**Selectivity**	**AmB selectivity / Compound selectivity**
**AmB**	0.25	8.5	34	1
**AmdeB**	>500	>500	-	-
**C2’deOAmB**	1	>500	>500	>14.7
**Compound**	**MIC-*C*. *albicans* (**μ**M)**	**MTC-Kidney cells (**μ**M)**		
**AmB**	0.25	2.4	34	1
**AmdeB**	>500	>80	0.16	0.005
**C2’deOAmB**	1	>80	>80	>2.35
**Preobrazhenskaya M.** [29]				
**Compound**	**MIC**_**50**_**-*Candida albicans* (10231) (**μ**g/ml)**	**HE**_**50**_**- Erythrocytes (**μ**g/ml)**	**Selectivity**	**AmB selectivity / Compound selectivity**
**AmB**	0.11	7.24	65.81	1
**D06**	0.08	100	1250	19
**A21** [30]				
**Compound**	**MIC**_**50**_**-*Candida albicans* (10231) (**μ**g/ml)**	**HE**_**50**_**- Erythrocytes (**μ**g/ml)**	**Selectivity**	**AmB selectivity / Compound selectivity**
**AmB**	0.20	6.7	33.5	1
**A21**	0.28	409	1460	43
**Compound**	**MIC**_**50**_**-*Candida albicans* (10231) (**μ**g/ml)**	**MIC**_**50**_**-kidney cells (293Q) (**μ**g/ml)**	**Selectivity**	**AmB selectivity / Compound selectivity**
**AmB**	0.20	9.7	48.5	1
**A21**	0.28	>500	1785	>36

We have introduced a novel AmB derivative with considerably increased safety in the treatment of mycosis (almost 7-fold via IP), and equal efficacy. A considerable advantage of the increased solubility of **A21** is that it can be delivered in aqueous or PBS solution. Moreover, even the highest dose applied in the acute toxicity study could be delivered just in aqueous solution. This could result in a better antibiotic for medical use in the treatment of fungal infections that are a growing health risk [69] and in the treatment of other ailments for which AmB has been proposed, e.g. protozoa and neoplastic cells.

## Abbreviations

AmB: Amphotericin B
DMPC: 1,2-dimyristoyl-*sn*-glycero-3-phosphocholine
DMSO: dimethylsulfoxide
POPC: 1-palmitoyl-2-oleoyl-sn-glycero-3-phosphocholine
dec: decomposed
HH: Head to Head
HT: Head to Tail
P: parallel
A: anti-parallel
PI: propidium iodide
μMol: micro Molar
FC_50_: fungicide concentration 50%
CFU: colony-forming units
HE_50_: hemolisis concentration 50%
TD_50_: toxic dose 50%
LD_50_: lethal dose 50%
MIC_50_: minimal inhibitory concentration 50%
fS: femto Siemens
μg/ml: micro gram per mililiter
ps: pico second
pA: pico Ampere
mV: mili Volt
SOCl_2_: thionyl chloride
MeOH: methanol
PyBOP: benzotriazol-1-yl-oxytripyrrolidinophosphonium hexafluorophosphate
Et_3_N: triethylamine
MTBE: methyl tertiary butyl ether
rt: room temperature
D: Dalton
IP: intraperitoneal
IV: intravenous
rmsd: root mean square deviation
